# Immunoregulation and anti*-*metalloproteinase bioactive injectable polysalicylate matrixgel for efficiently treating osteoarthritis

**DOI:** 10.1016/j.mtbio.2022.100277

**Published:** 2022-05-06

**Authors:** Xinlin Jia, Junping Ma, Xuzhuo Chen, Wentao Li, Xianhao Zhou, Bo Lei, Xin Zhao, Yuanqing Mao

**Affiliations:** a Shanghai Key Laboratory of Orthopedic Implant, Department of Orthopedic Surgery, Shanghai Ninth People's Hospital, Shanghai Jiao Tong University School of Medicine, Shanghai 200011, China; b Key Laboratory of Shaanxi Province for Craniofacial Precision Medicine Research, College of Stomatology, Frontier Institute of Science and Technology, Xi'an Jiaotong University, Xi'an, China; c Department of Oral Surgery, Shanghai Ninth People's Hospital, Shanghai Jiao Tong University School of Medicine, Shanghai Key Laboratory of Stomatology & Shanghai Research Institute of Stomatology National Clinical Research Center of Stomatology, Shanghai 200011, China

**Keywords:** Bioactive materials, Multifunctional hydrogel, Tissue engineering, Osteoarthritis

## Abstract

Current treatments of osteoarthritis, such as oral medication and intra-articular injections, only provided temporary relief from pain and achieved limited advance in inhibiting progression. The development of new treatments is hindered by the complicated and unclear pathological mechanisms. Oxidative stress and immune inflammation are believed to be the important factors in the induction and progression of osteoarthritis. Herein, this work presents a bioactive material strategy to treat osteoarthritis, based on the FPSOH matrixgel with robust anti-inflammatory activity through inhibiting the oxidative stress and nuclear factor kappa B signaling, preventing the metalloproteinase, as well as inducing M2 polarization of macrophage, thereby providing immune regulation of synovial macrophages and suppressing the progression of synovitis and osteoarthritis. *In vivo* experiments demonstrated that FPSOH hydrogel can prevent papain-induced osteoarthritis and its progression, and provide dual protection for cartilage and synovium, as compared with commercial sodium hyaluronate.

## Graphical abstract

**Figure:**
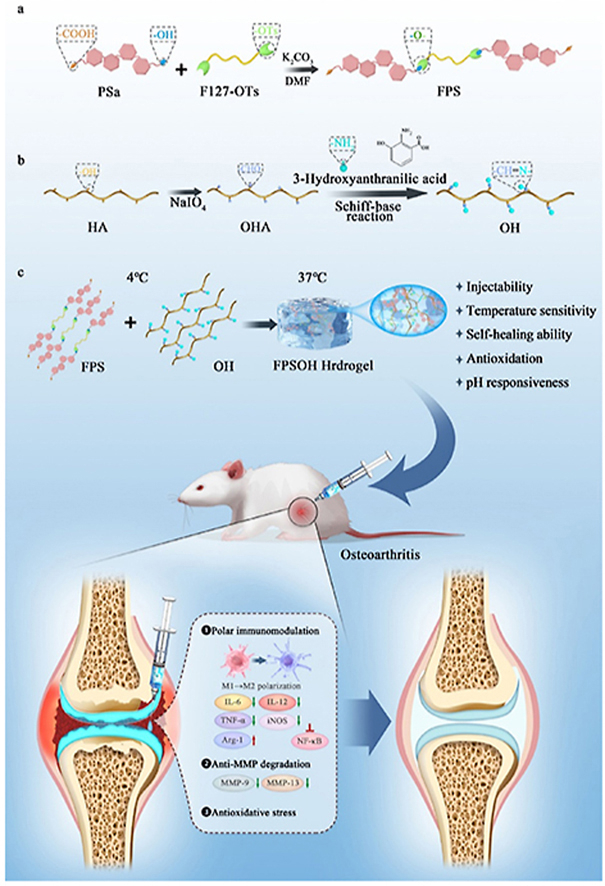

## 1 Introduction

Osteoarthritis is the most common degenerative disease of the joints seen in clinical practice. It is caused by many factors, such as aging, injury, autoimmune diseases, and abnormal metabolism [1,2]. Patients with osteoarthritis experience a range of symptoms, including joint pain, swelling, stiffness, and limited mobility, which can seriously affect their life and lead to disability. Previous studies have revealed that the pathological features of osteoarthritis include progressive cartilage degeneration, subchondral bone remodeling, and osteophyte formation, but the underlying mechanisms are not yet understood [2]. Most studies support the theory that oxidative stress plays an important role in the progression of osteoarthritis [[3], [4], [5]]. Oxidative stress can cause the degeneration and apoptosis of chondrocytes as well as degradation of the cartilage matrix, which may expose the chondrocytes and the protective effect of the matrix is lost. Furthermore, increasing evidence suggests a strong correlation between synovitis and the pathology and progression of osteoarthritis [6]. Osteoarthritis is often accompanied by the inflammatory hyperplasia of the synovial tissue and causes significant changes, such as the activation of synovial macrophages [7,8]. Synovial macrophages are activated and differentiated into M1 (pro-inflammatory) and M2 (anti-inflammatory) phenotypes when they are stimulated by changes in the microenvironment [9]. M1 macrophages secrete a large number of pro-inflammatory factors, such as IL-6, TNF-α, and IL-1β, which promote inflammation [6,10,11]. In contrast, M2 macrophages secrete anti-inflammatory factors, such as IL-10 and Arg-1, which have anti-inflammatory and immunosuppressive effects [[10], [11], [12]]. As osteoarthritis progresses, most of the activated synovial macrophages are of the M1 phenotype. The pro-inflammatory factors secreted by these macrophages promote the proliferation of synovial tissue, but they also act on chondrocytes in the joints resulting in their degeneration and apoptosis. Ultimately, the combined effects of oxidative stress and inflammatory factors accelerate the progression of osteoarthritis and cause severe joint degeneration.

At present, clinical treatments for early osteoarthritis are based on oral non-steroidal anti-inflammatory drugs [13]. One constituent of these drugs is salicylic acid, which has good anti-inflammatory, antioxidant, and analgesic effects [[14], [15], [16]]. Salicylic acid has also been used in the treatment of human skin diseases and arthritis owing to its good biocompatibility and biological activity [[17], [18], [19]]. Furthermore, polymers based on salicylic acid have shown potential in many applications. For example, poly-salicylic acid nanoparticles are used for the delivery of miRNA in gene therapies for inflammatory diseases in animals [20]. In another treatment, hyaluronic acid gel is injected to the joint cavity to offer relief from and intervene in the progression of osteoarthritis [[21], [22], [23]]. Hyaluronic acid is a glycosaminoglycan that helps to protect joint cartilage, transport nutrients, lubricate joints, and cushion vibrations, and within joints its synthesis and metabolism are strictly regulated to maintain a dynamic balance [24,25]. However, the concentration, elasticity, and viscosity of hyaluronic acid are reduced in the synovial fluid of patients with osteoarthritis [[26], [27], [28]]. Thus, there is less protection for the articular cartilage, which is exposed to the inflammatory environment and begins to degenerate [29]. Now, there are a variety of intra-articular injection products that use hyaluronic acid and its derivatives for the treatment of osteoarthritis [23,25,27]. However, such treatments do not completely prevent the progression of arthritis nor the proliferation of synovial tissue. Hence, injections of hyaluronic acid gel to the joint cavity only offer a temporary improvement of symptoms. Therefore, hydrogels that are anti-inflammatory, antioxidant, and capable of regulating synovial macrophage polarization are expected to form the next generation of treatments for osteoarthritis.

**Scheme 1:**
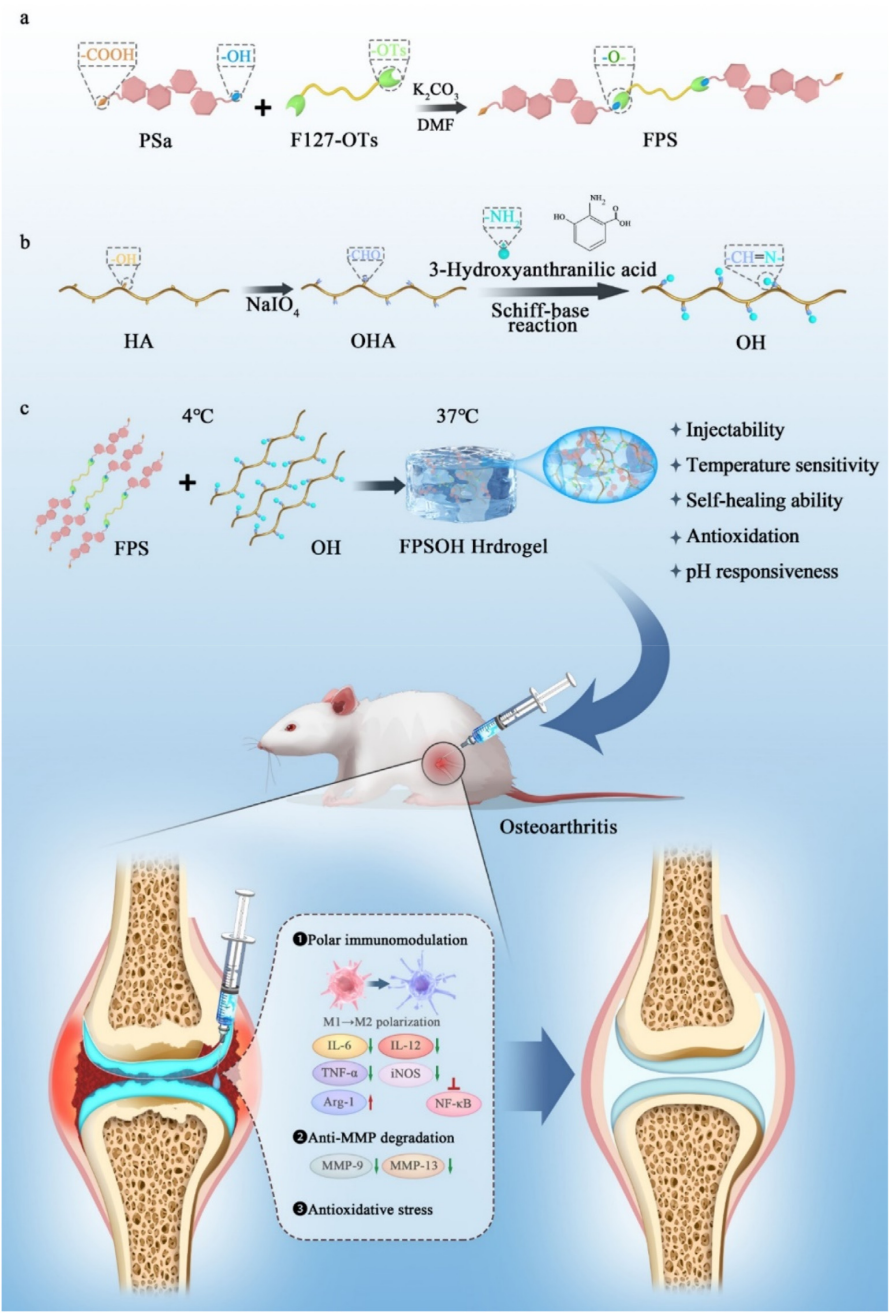
Schematic illustration of synthesis of FPSOH hydrogel and treatment of osteoarthritis.

This study intends to develop a multifunctional hydrogel (FPSOH) with robust anti-inflammatory activity through inhibiting the oxidative stress and nuclear factor kappa B signaling, preventing the metalloproteinase, as well as inducing M2 polarization of macrophage, thereby providing immune regulation of synovial macrophages and suppressing the progression of synovitis and osteoarthritis. FPSOH hydrogel was fabricated through a self-crosslinking between poly (salicylic acid)-F127-poly (salicylic acid) (FPS) and hyaluronic acid-3-hydroxyanthranilic acid (OH) (Scheme 1). OH was synthesized *via t*he Schiff base reaction between aldehyde-HA and 3-HAA, which is responsive to pH changes [30]. FPS is a temperature-sensitive hydrogel and possessed good antiinflammation/antioxidation activity in tisssue repair, which was reported by our group recently [31]. 3-HAA is a metabolite produced by the metabolism of the essential amino acid tryptophan, which has not been discussed in any studies on the treatment of osteoarthritis [[32], [33], [34], [35]]. Nevertheless, it can reduce inflammation by regulating the conversion from M1 to M2 macrophages [[36], [37], [38]]. Furthermore, 3-HAA can inhibit nuclear factor kappa B (NF-κB) and other classic inflammatory signal pathways as well as the expression and release of inflammatory factors, so it has a good anti-inflammatory effect [33,37,39,40]. In this work, we investigate the effects of FPSOH hydrogel with anti-inflammatory/antioxidant/*anti*-metalloproteinase on synergistic treatment of osteoarthritis and synovitis.

## 2 Materials and methods

### 2.1 Synthesis and characterizations of FPSOH hydrogel

The synthesis of PSa (poly salicylic acid), FPS (F127-PSa) and OHA (aldehyde hyaluronic acid) was according to the previous work [31,41]. The detailed procedures were shown in the supporting file. The OH (OHA@3-HAA) was synthesized by the Schiff base reaction between the aldehyde in OHA and the amino in 3-HAA (99.38%, Macklin). Specially, 100 ​μg 3-HAA was ultrasonically dispersed in 1 ​mL of deionized water, and 30 ​mg OHA was added for further 30 ​min reaction. The final FPSOH hydrogel (poly salicylic acid-F127-poly salicylic acid (FPS) and aldehyde hyaluronic acid@3-hydroxyanthranilic acid (OH)) was made by mixing 300 ​mg FPS and OH. The above mixture was placed at 4 ​°C to form a uniform sol and formed the hydrogel at 37 ​°C. The physicochemical properties and morphological structure were characterized by ^1^H nuclear magnetic resonance (^1^H NMR) instrument (Ascend 400 ​MHz, Bruker), fourier transform infrared spectroscopy (FTIR, BRUKER OPTIK GmbH, Rudolf Plank Str. 23, D-76275 Ettlingen, Germany), Nanodrop (Schatzbogen 52 D-81829 Munich, Germany) and scanning electron microscopy (SEM, Quanta 250 FEG, FEI).

### 2.2 Multifunctional properties evaluations

A camera was applied to observe the thermal sensitivity, injectability, along with self-healing performance of FPSOH. A TA rheometer (DHR-2) was employed to explore the rheological property of FPS, FPSO, and FPSOH hydrogels. In vitro antioxidant capability of hydrogels was evaluated through the 1,1-diphenyl-2-picrylhydrazyl (DPPH) method. At the same time, degradation and sustained 3-HAA release behavior of FPSOH hydrogel in PBS were tested. The details are shown in Section S1.3 of SI.

### 2.3 Rat osteoarthritis model

The animal experiments in this study were approved by the Animal Research Committee of Ninth People Hospital, Shanghai Jiao Tong University (SH9H-2021-A32-1). Seventy-six-week-old male SD rats were selected and randomly divided into seven equal groups: the control, papain-induced osteoarthritis, 3-HAA, FPS, FPSO, FPSOH, and sodium hyaluronate (SH) treatment group (10 rats in each group). The rats were weighed and 1% pentobarbital (40 ​mg ​kg^−1^) was injected intraperitoneally for anesthesia. Subsequently, the hair around the right knee joint was removed and the area was disinfected with iodophor. For the control group, 50 ​μL of normal saline was injected into the right knee joint cavity; for the remaining groups, 50 ​μL of 8% papain was injected into the knee cavity, and this was repeated on the third and fifth days. Starting from the sixth day, the corresponding solutions/hydrogels were injected into the joint cavity on a weekly basis for treatment. In the third and sixth weeks after the treatment, the knee joints and corresponding synovium were removed, placed in 4% paraformaldehyde for 48 ​h, and then used for radiological and histological analyses.

### 2.4 Osteoarthritis therapy evaluations

Micro-CT scans were performed with a high-resolution micro-CT (Skyscan1176, USA Bruker Siemens Inveon, Eschborn, Germany). The scanning resolution was 9 ​μm, the source voltage was 50 ​kV, and the source current was 500 ​μA.

After the micro-CT scans were performed, the knee joint samples were soaked in 10% ethylenediaminetetraacetic acid (EDTA; pH ​= ​7.4) to decalcify for 4–6 weeks. The knee joint samples were then embedded in paraffin and sliced in the sagittal plane into tissue sections 4 μm thick for HE, safranine O-fast green, TRAP, TUNEL, and immunohistochemical staining. In addition, CD68, CD206, and iNOS were used to complete the immunohistochemical staining of the synovial tissue. The staining results were confirmed under a microscope, and the resulting pictures were analyzed using the Image J software for statistical analysis.

### 2.5 Statistical analysis

The data are expressed as the mean ​± ​standard deviation (SD), and each group of experiments was repeated independently three times. Analysis of variations (ANOVA) was used for comparison, and the GraphPad Prism 8.0 (La Jolla, CA, USA) software was used for statistical analysis. ANOVA and Tukey multiple comparison tests were used for group comparisons. The statistical significance was expressed as ∗P ​< ​0.05, ∗∗P ​< ​0.01, or ∗∗∗P ​< ​0.001.

## 3 Results and discussion

### 3.1 Physicochemical structure characterizations

**Fig. 1:**
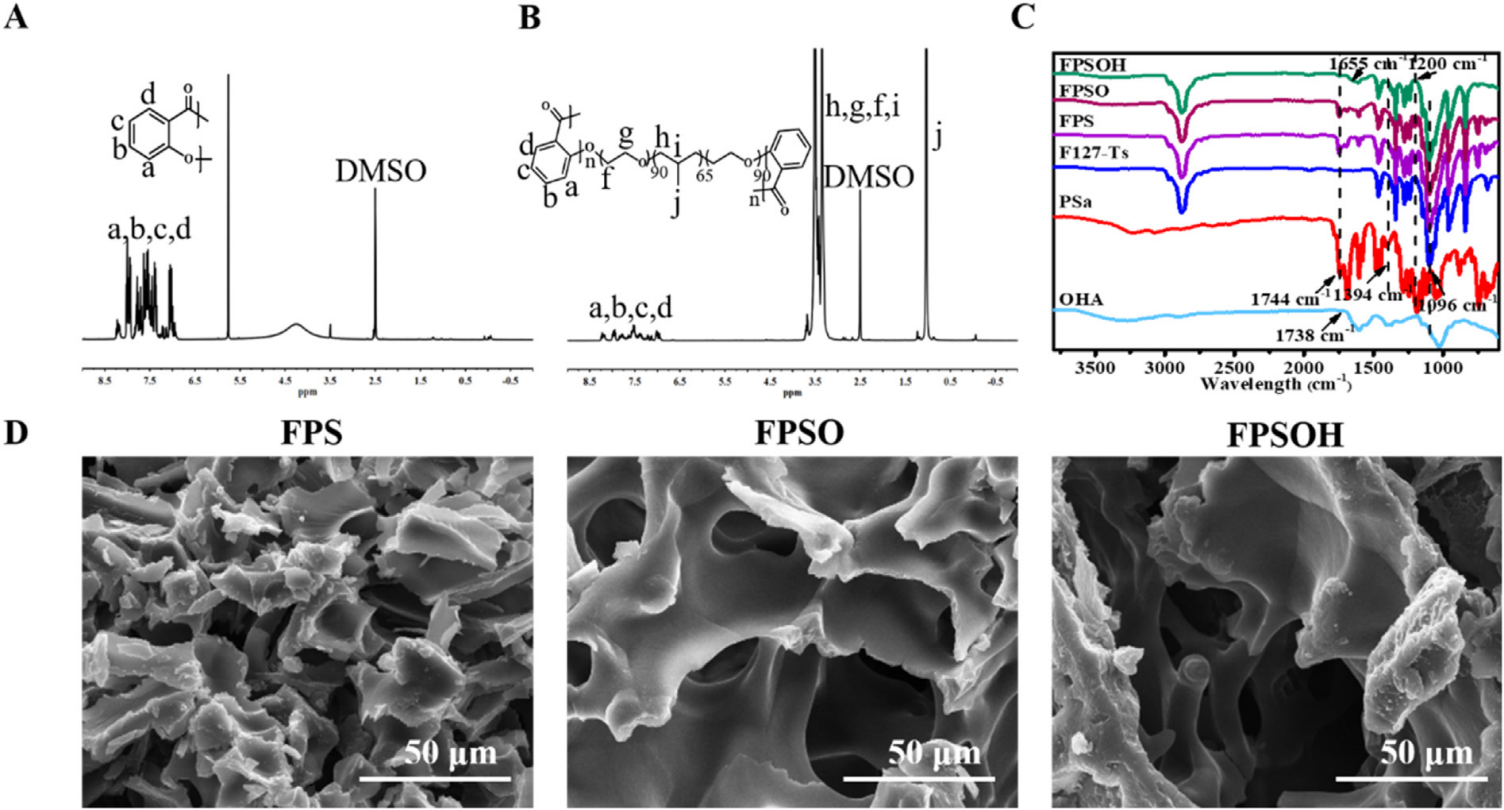
**Physicochemical structure characterizations.**^1^H NMR spectra of PSa (A) and FPS (B); (C) FTIR spectra of various polymers; (D) SEM images of various hydrogels.

Fig. 1 displays the physicochemical properties and special microstructure of hydrogels. The chemical structure and characteristic function groups of samples were identified by ^1^H NMR and FTIR analysis to confirm the successful synthesis of the polymers. In Fig. 1A, the peaks at 8.30–6.85 ​ppm were defined as protons from the benzene ring (–C_6_H_4_–) belonging to PSa polymer. The ^1^H NMR spectrum of FPS is shown in Fig. 1B, in which the new peak at 1.04 ​ppm was corresponded to the hydrogen on methyl group (CH_3_-) of F127, except for the peaks belonging to H on –C_6_H_4_- in PSa [31]. The characteristic function groups of the polymers were further determined by FTIR instrument (Fig. 1C). The absorption peak found at 1738 ​cm^−1^ was assigned to the aldehyde group (-CHO) stretch from OHA, which verified that HA was successfully oxidized [41]. The appearance of ester bond peak at 1744 ​cm^−1^ indicated that Sa was polymerized to form PSa. The absorption peak at 1394 ​cm^−1^ in the FTIR spectrum of PSa was attributed to unreacted phenolic hydroxyl (-OH) at the chain end of PSa. The absorption peak caused by unique ether bond in F127-OTs was found at 1096 ​cm^−1^. In contrast to pure PSa and F127, the simultaneous presence of the peaks at 1108 ​cm^−1^ and 1744 ​cm^−1^ manifested that FPS was composed of F127 and PSa. The successful grafting of PSa onto F127 was demonstrated by the existence of the peak assigned as aromatic ether bond at 1200 ​cm^−1^ and the disappearance of absorption peak representing phenolic hydroxyl at 1394 ​cm^−1^ [31]. In the FTIR spectral curve of freeze-dried FPSOH sample, a new band appeared at 1655 ​cm^−1^ (-C=N-) and the peak at 1738 ​cm^−1^ (-CHO) vanished, indicating the occurrence of Schiff base reaction between the aldehyde group of OHA and –NH_2_ from 3-HAA [42,43]. The microstructure of hydrogels was observed by SEM. FPS, FPSO and FPSOH hydrogels exhibited significant three-dimensional porous morphology (Fig. 1D). Compared with FPSO, FPSO and FPSOH possessed larger pore size and more complete pore structure, revealing morphology stability had been improved to some extent.

### 3.2 Multifunctional physicochemical properties

**Fig. 2:**
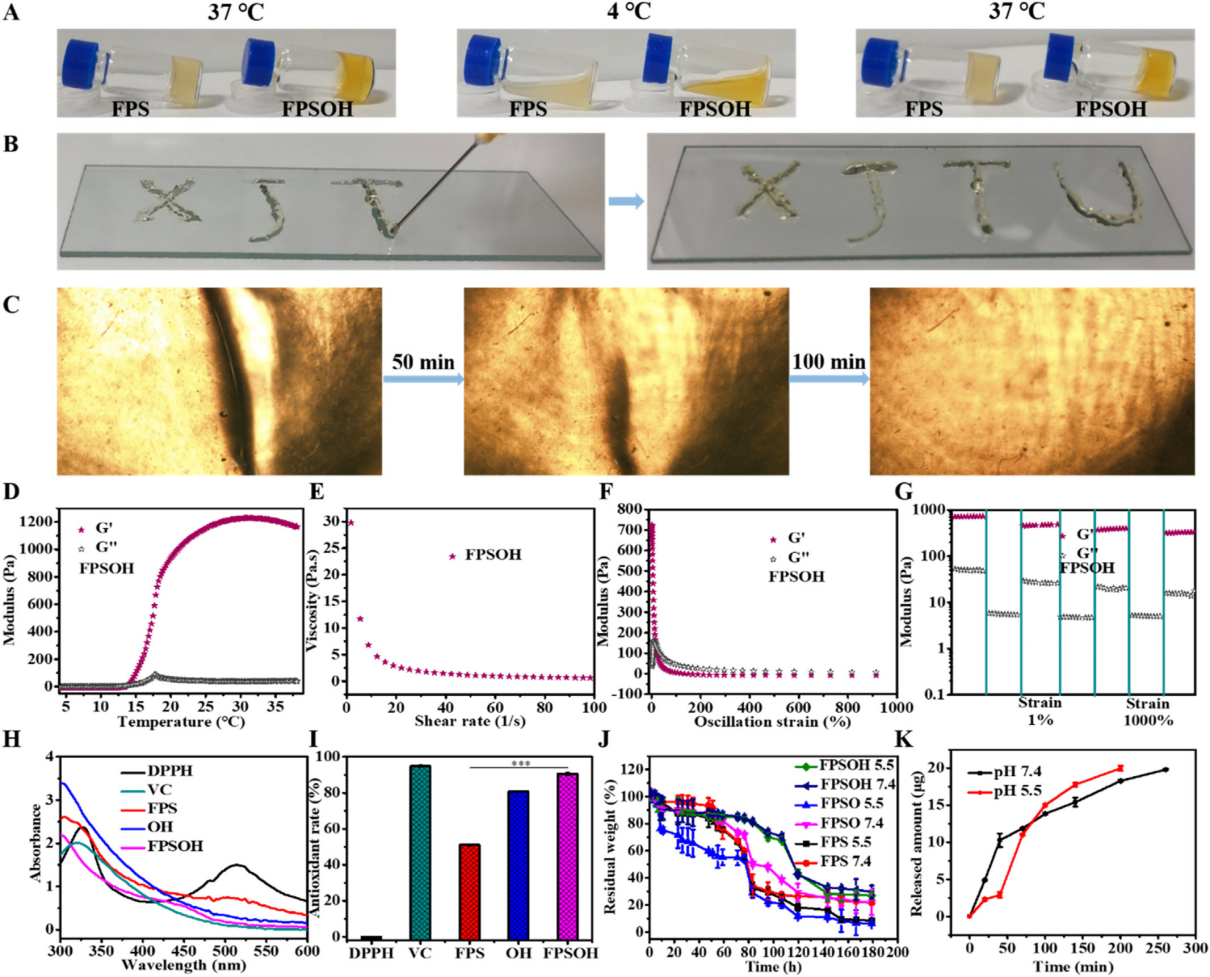
**Multifunctional physicochemical properties.** Optical photograph of (A) sol-gel transition with temperature change and (B) the injectability of FPSOH hydrogel. (C) Self-healing behavior of FPSOH hydrogel. (D) G′ and G″ with temperature change; (E) Viscosity versus shear rate; (F) G′ and G″ from 1 to 1000% oscillation strain; (G) G′ and G″ change with cyclic strain of FPSOH hydrogel. (H) UV–vis spectra of DPPH, VC, FPS, OH, and FPSOH. (I) Antioxidant rate of VC, FPS, OH and FPSOH. (J) The residual weight of FPS, FPSO and FPSOH hydrogels placed in PBS with different pH 5.5 and 7.4 for a specific time. (K) The released behavior of 3-HAA from FPSOH hydrogel in PBS solution with different pH.

The heat sensitivity, injectability and self-healing ability of hydrogels were investigated. Fig. 2A displays the invertible sol-gel transition capabilities of hydrogels. The FPSOH hydrogel kept in a gel state at 37 ​°C, and turned into a liquid state when cooled to 4 ​°C. When the temperature advanced to 37 ​°C, it showed a gel state again, showing superior temperature sensitivity. At room temperature, the FPSOH could be smoothly injected into a specific pattern by syringe, such as “XJTU” without clogging (Fig. 2B), proving its preeminent injectability. The crack on the cut hydrogel could be recovered rapidly after different times, suggesting their good self-healing ability (Fig. 2C). The rheological testing under different conditions were conducted to appraise the mechanical behavior of FPS, FPSO, FPSOH hydrogels. Fig. 2D shows the changes in the storage modulus (G′) and loss modulus (G″) of the hydrogels with temperature, it was found that the G′ and G″ gradually augmented from temperature increases with the temperature rising from 4 ​°C to 37 ​°C. It was worth noting that the G′ of FPSOH gradually became higher than G″ at 13.71 ​°C. With the growth of shear rate, the viscosity of FPSOH hydrogel exhibited a rapid decline, manifesting that hydrogel held the characteristics of shear thinning, which was the premise of its injectability (Fig. 2E). The enhancement of the oscillating strain caused the G″ of FPSOH hydrogel to be gradually lower than G′, which was also the evidence of the shear thinning ability of the hydrogel (Fig. 2F). The self-healing ability of hydrogels was assessed by repeatedly measuring G′ and G″ in three cycles of 1% and 1000% (Fig. 2G). After three cycles, although the recovery of G′ and G “of FPSOH had some loss under 1% shear strain, FPSOH still wielded remarkable self-healing performance. The similar mechanical behavior was observed in the rheological characterization of FPS and FPSO hydrogels (Figure S1 in SI). Based on its versatility, heat sensitivity, injectability and self-healing, FPSOH hydrogel hold great potential in treating osteoarthritis.

### 3.3 Antioxidation, biodegradation and sustain release evaluation

The antioxidant, biodegradation, and sustain release were described in Fig. 2H–K and Figures S2. The DPPH method to determine the *in vitro* antioxidant capacity of hydrogels was adopted. The UV–vis spectral curves showed that untreated DPPH had a high UV–vis absorption value at 515 ​nm, while the absorption values of FPS, OH and FPSOH at 515 ​nm decreased sequentially. Especially under the synergistic effect of FPS and OH, the antioxidant effect of FPSOH was significantly enhanced (Fig. 2H). In addition, the statistical data revealed that the antioxidant rate of FPSOH sample was as high as 90.56%, which was comparable to 94.88% of commercially available ascorbic acid (VC). This mainly gave the credit to the fact that FPSOH hydrogel contained masses of active functional groups (Fig. 2I). As displayed in Figure S2, contrasted to the darker color of solution after FPS and OH treatment, the color of solution treated by FPSOH and VC was bright yellow, and the elimination of free radicals was in-depth certified. The weight change of hydrogel samples added with PBS (pH 7.4 and 5.5) was detected to evaluate the biodegradability of hydrogel (Fig. 2J). After 180 ​h, the remaining weight of all groups was less than 30%. The weight loss of FPSO (93.76% at pH 5.5, 78.10% at pH 7.4) is similar to that of FPS (91.46% at pH 5.5, 78.36% at pH 7.4). In comparison, FPSOH had a smaller weight loss (73.06% at pH 5.5, 70.25% at pH 7.4). This phenomenon might be because the second layer network formed by OHA and 3-HAA, which strengthened the stability of hydrogel. The slow degradation of FPSOH hydrogel can play a role in the continuous treatment of osteoarthritis. The trend of 3-HAA release from FPSOH was shown in Fig. 2K. The FPSOH soaked in pH 5.5 and 7.4 ​PBS almost completely released the 3-HAA after 200 ​min and 260min respectively. This rapid release result would provide timely and effective treatment for acute inflammation.

### 3.4 Cytotoxicity, anti-inflammation and macrophage polarization *in vitro*

**Fig. 3:**
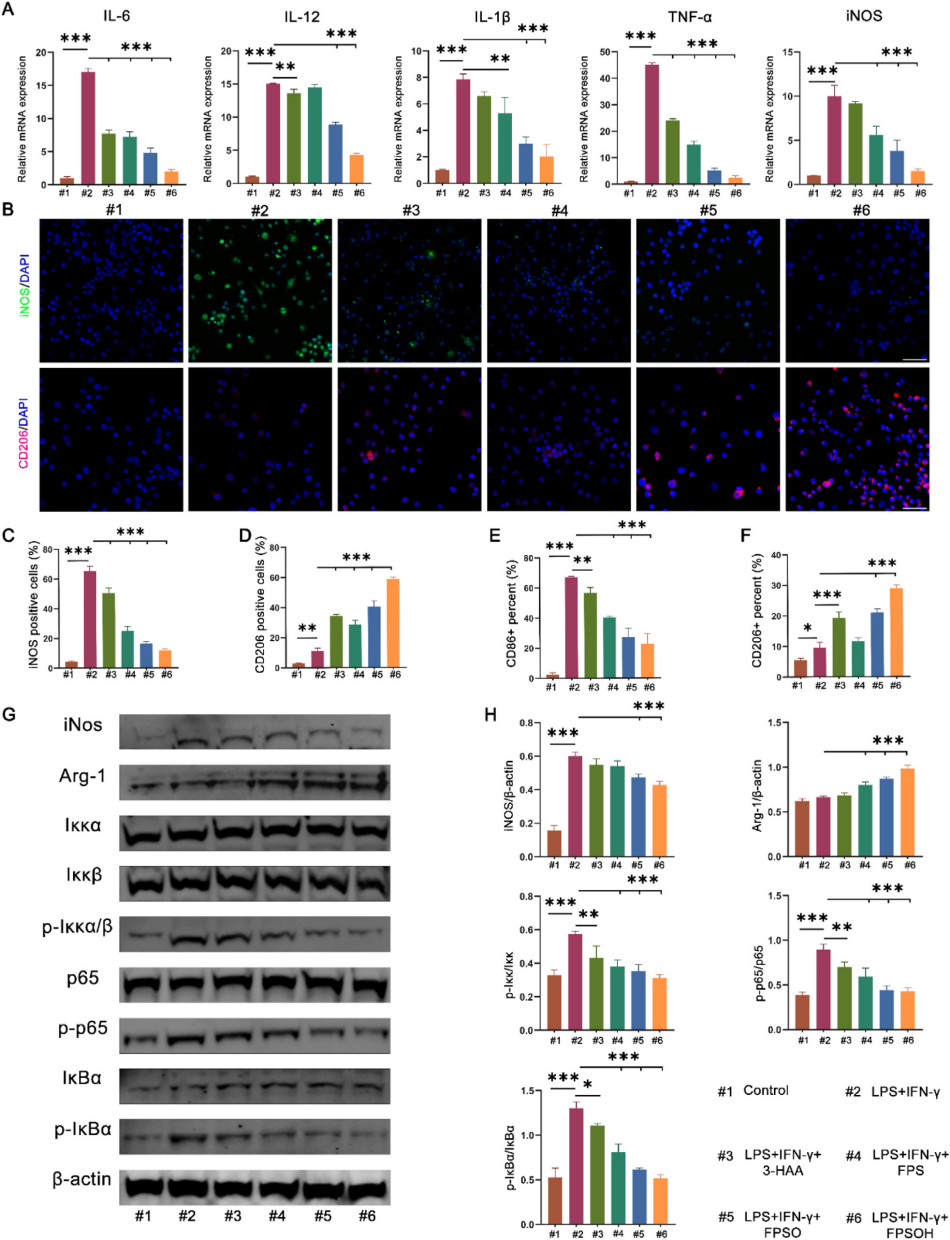
**Effect of FPSOH hydrogel on anti-inflammation and macrophage polarization.** (A) Expression of M1-ralated genes analyzed by RT-qPCR in RAW 264,7 macrophages. (B) Confocal images of M1 and M2 macrophages co-cultured with FPSOH. iNOS was stained for marking M1 macrophages. CD206 was stained for marking M2 macrophages. Scale bar is 50 ​μm. (C) and (D) Percentage of positive cells in confocal images of iNOS and CD206. (E) and (F) Flowcytometry analysis of macrophages treated by FSPOH in the presence of LPS plus IFN-γ for 24 ​h. (G) and (H) Western blotting displaying the expression of inflammatory-related proteins and the activation of NF-κB pathways with the indicated treatment. *∗P* ​< ​0.05; *∗∗P* ​< ​0.01; *∗∗∗P* ​< ​0.001.

First, the biocompatibility of FPS, FPSO, and FPSOH hydrogels with rat chondrocytes was tested by Live/Dead cell staining. After three days of co-cultivation, there were no statistical differences in the cell growth of each group (Figure S3 in SI), indicating that the hydrogels and chondrocytes had good biocompatibility. Then, the effects of each hydrogel on the inflammation of RAW264.7 macrophages were investigated using RT-qPCR test. The mRNA expression of the pro-inflammatory genes IL-6, IL-12, IL-1β, TNF-α and iNOS increased significantly under co-stimulation with LPS and IFN-γ compared with that in the control group (Fig. 3A). In contrast, the expression of these inflammatory genes was significantly reduced after co-cultivation with the 3-HAA and FPS, FPSO, or FPSOH hydrogels. Compared with FPSO, FPS and drug group, FPSOH group showed higher inflammatory inhibition, which indicated that FPSO and drug had a good synergistic therapeutic effect.

In addition, immunofluorescence staining and flow cytometry were used to investigate the effects of the hydrogels on the polarization of macrophages. The expression of iNOS (the M1 macrophage marker) increased significantly in the state of inflammation induced by LPS and IFN-γ (Fig. 3B–D and S4). However, the expression of iNOS was reduced by different degrees in each treatment group after co-cultivation with the hydrogels. This reduction was particularly significant in the FPSOH group, where the inhibition of iNOS expression was considerably stronger than that in the 3-HAA group. The FPSO and FPS groups also inhibited iNOS expression. Moreover, the expression of the cluster of differentiation CD206 (M2 macrophages marker) showed the opposite trends. The expression of CD206 in the LPS plus IFN-γ stimulated group was significantly lower than that of the 3-HAA, FPS, FPSO, and FPSOH groups (Fig. 3D). To further verify that the hydrogels could regulate the conversion from M1 to M2 macrophages, CD86 and CD206 were used as markers for M1 and M2 macrophages [10], respectively, and were detected using flow cytometry. As shown in Fig. 3E, the proportion of CD86 positive cells (M1 macrophages) in the LPS plus IFN-γ stimulated group compared to the control was 67.13 ​± ​0.60% and those for the 3-HAA, FPS, FPSO, and FPSOH groups were 56.63 ​± ​3.62%, 40.43 ​± ​0.72%, 27.33 ​± ​5.77% and 22.73 ​± ​6.79%, respectively. Moreover, the proportion of CD206 positive cells in the LPS plus IFN-γ simulated group compared to the control was 9.60 ​± ​1.75% and those for the 3-HAA, FPS, FPSO, and FPSOH groups after treatment were 19.30 ​± ​1.97%, 11.60 ​± ​1.30%, 21.00 ​± ​1.25% and 29.03 ​± ​0.97%, respectively (Fig. 3F). These results indicate that the hydrogels can inhibit the expression of M1 macrophages and regulate the polarization of macrophages to M2 macrophages.

Further, to explore the effects of the hydrogels on the inflammatory signal pathway and the expression of macrophage polarization-related proteins, Western blot (WB) tests were used to detect changes in the expression of relevant proteins. The effects of the hydrogels on the NF-κB signaling pathway, a classic inflammatory response signaling pathway, were investigated [[10], [11], [12]]. As shown in Fig. 3G–H, the phosphorylation of p65, Iκκα/β, and IκBα was activated under the stimulation of LPS plus IFN-γ. However, the phosphorylation levels were inhibited to varying degrees after co-cultivation with the hydrogels. This inhibitory effect was most significant in the FPSOH group, followed by the FPSO, FPS, and 3-HAA groups. This shows that FPSO and the 3-HAA have a good synergistic effect. In addition, the expression of iNOS showed a decreasing trend after co-cultivation with the hydrogels, and the expression of Arg-1 was regulated upwards. This shows that the hydrogels can inhibit the activation of the NF-κB signaling pathway, thereby inhibiting inflammation, and can affect protein changes related to the polarization of macrophages, thus regulating the polarization of macrophages.

### 3.5 Anti-oxidative stress, anti-apoptosis effects and antidegradation effect *in vitro*

Articular cartilage is mainly composed of chondrocytes and cartilage matrix. On the one hand, the cartilage matrix protects the chondrocytes and on the other hand, as the active centers of articular cartilage, the chondrocytes control the anabolism and catabolism of the cartilage matrix [44]. Collagen II and aggrecan are secreted by normal chondrocytes, which are important components of the cartilage matrix. In the pathological process of osteoarthritis, chondrocytes undergo hypertrophy due to oxidative stress and inflammatory factors [45]. Hypertrophic chondrocytes express collagen 10 and secrete a series of matrix metalloproteinases, such as MMP9 and MMP13, which decompose the cartilage matrix, so the chondrocytes lose the protection of the cartilage matrix [44,46]. Further exposure to oxidative stress and inflammation from the microenvironment causes a more intense degeneration of the chondrocytes and the progression of osteoarthritis. Furthermore, the excess of reactive oxygen species (ROS) produced by oxidative stress can lead to apoptosis of chondrocytes. Previous studies have reported that H_2_O_2_ can stimulate oxidative stress in chondrocytes and produce a large number of ROS, which results in cartilage apoptosis and degeneration. Thus, this study used H_2_O_2_ to simulate the oxidative stress of chondrocytes during the pathogenesis of osteoarthritis.

**Fig. 4:**
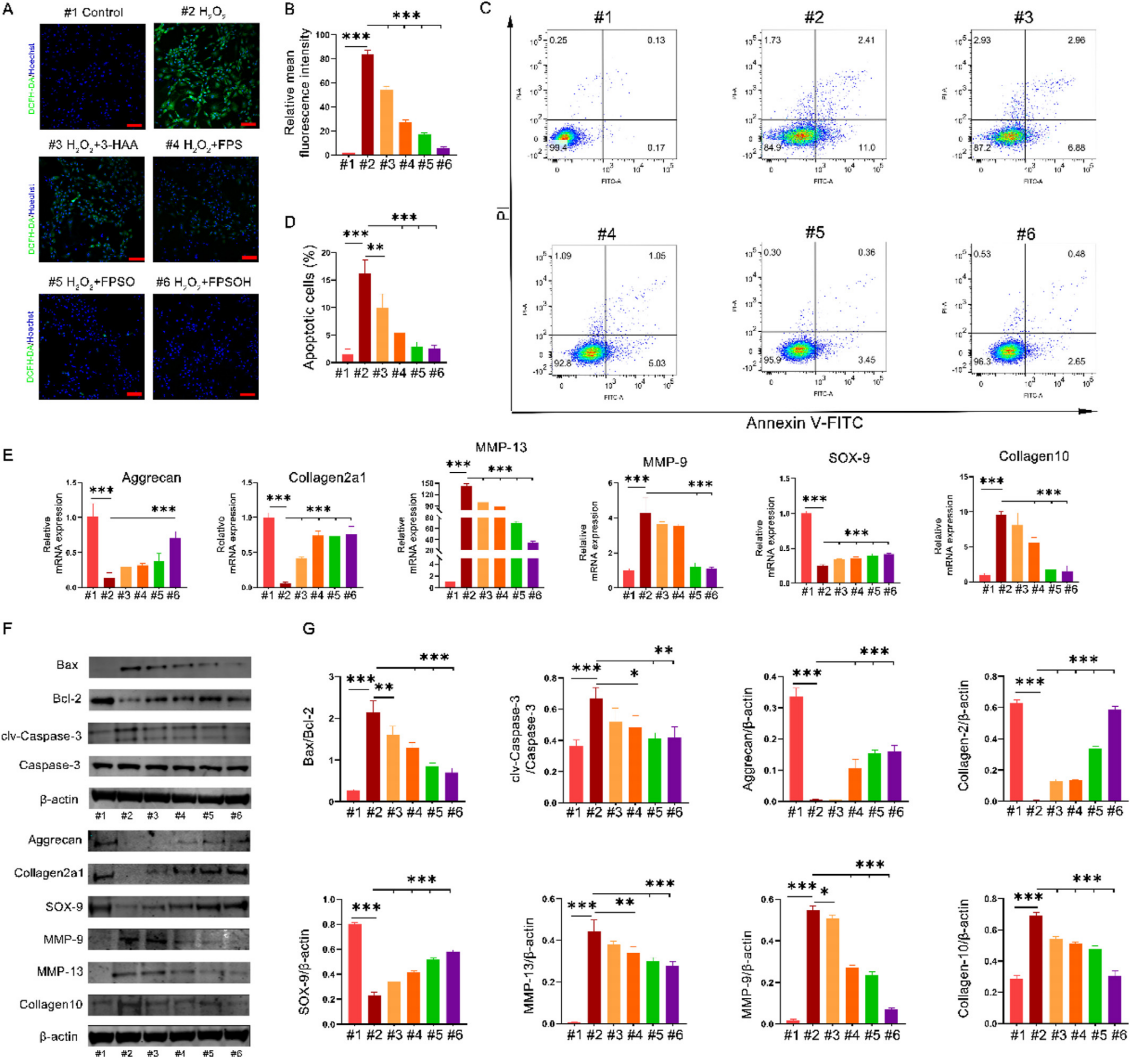
**Protective effect of FPSOH hydrogel on chondrocytes.** (A) and (B) Scavenging of cellular ROS. Scale bar is 100 ​μm. (C) and (D) Apoptosis rates for H_2_O_2_-induced chondrocytes were analyzed by flow cytometry with Annexin V-FITC/PI analysis. (E) The expression of genes related to cartilage metabolism was analyzed by RT-qPCR. (F) and (G) The expression of apoptosis related proteins and metabolism related proteins in chondrocytes with the indicated treatment. *∗P* ​< ​0.05; *∗∗P* ​< ​0.01; *∗∗∗P* ​< ​0.001.

After treatment, a 2′,7′-dichlorofluorescein diacetate (DCFH-DA) probe was used to detect the quantity of ROS in each group. As shown in Fig. 4A–B, the 3-HAA, FPS, FPSO, and FPSOH groups demonstrated inhibitory effects on oxidative stress compared to the H_2_O_2_-treated group, which showed that ROS fluorescence intensity decreased gradually. Further quantitative analysis by flow cytometry showed that the number of ROS-positive cells decreased significantly after the treatment of hydrogels (Figure S5). These results indicate that the hydrogels can inhibit the oxidative stress of chondrocytes induced by H_2_O_2_. Chondrocytes also undergo apoptosis under oxidative stress. The anti-apoptosis effects of the different hydrogels were investigated using flow cytometry. As shown in Fig. 4C–D, under H_2_O_2_ treatment, chondrocyte apoptosis increased significantly. In contrast, the number of apoptotic cells were significantly reduced between groups, especially in the FPSOH group.

To further investigate the protective effects of the hydrogel on chondrocytes under oxidative stress, we detected the expression of genes and proteins related to cartilage synthesis and catabolism. The expression of chondroprotective genes, such as *SOX-9*, *Collagen2a1* and *Aggrecan*, was significantly reduced compared with that in the control group under H_2_O_2_ stimulation (Fig. 4E). In contrast, the results showed that the hydrogels inhibited the decline of the three chondroprotective genes to varying degrees in comparison to the H_2_O_2_-treated group. However, it is worth noting that although the 3-HAA, FPS, and FPSO groups inhibited the reduction in the expression of *SOX-9* and *Collagen2a1*, compared to the H_2_O_2_-treated group, none of them had a significant effect on the expression of *Aggrecan*. In contrast, the FPSOH group significantly inhibited the reduced expression of *Aggrecan*, showing that the 3-HAA and FPSO had good synergistic protective effect. The protective effects of the hydrogels on cartilage were investigated further through the detection of degenerative genes. As shown in Fig. 4E, the expression of cartilage catabolism-related genes, such as *MMP-9*, *Collagen 10* and *MMP-13*, increased significantly compared with that in the control group under the action of H_2_O_2_. By contrast, the expression of these genes was significantly inhibited under the co-cultivation of the FPSO and FPSOH groups compared to the H_2_O_2_-treated group. In addition, although FPS significantly inhibited the expression of *MMP-13* and *Collagen 10*, it had no significant inhibitory effects on *MMP-9*. In the 3-HAA group, only the expression of *MMP-13* was inhibited significantly, and no significant inhibitory effects on the expression of *MMP-9* or *Collagen 10* were observed. Furthermore, Fig. 4F–G shows that the hydrogels not only inhibited the expression of decomposition-related proteins and pro-apoptotic protein (Bax), but also increased the expression of protective proteins and anti-apoptotic protein (Bcl-2).

### 3.6 Therapeutic effect on papain-induced osteoarthritis in rats

To further demonstrate that the hydrogels can inhibit synovial inflammation and cartilage degeneration, a rat osteoarthritis model was established by an intra-articular injection of papain, and an intra-articular injection of hydrogel was used to treat the osteoarthritis. Many studies have reported that intra-articular injections of papain can induce knee osteoarthritis and cause inflammatory lesions in the synovial tissue of the joint [47,48]. Starting from the fifth day of the modeling, the 3-HAA only, FPS, FPSO and FPSOH hydrogels were injected to the joint cavity of the rats on a weekly basis. To better evaluate the therapeutic effects of hydrogels, an additional group was treated with injections of sodium hyaluronate (SH), a hydrogel that is used clinically for joint cavity injections to treat patients with osteoarthritis.

**Fig. 5:**
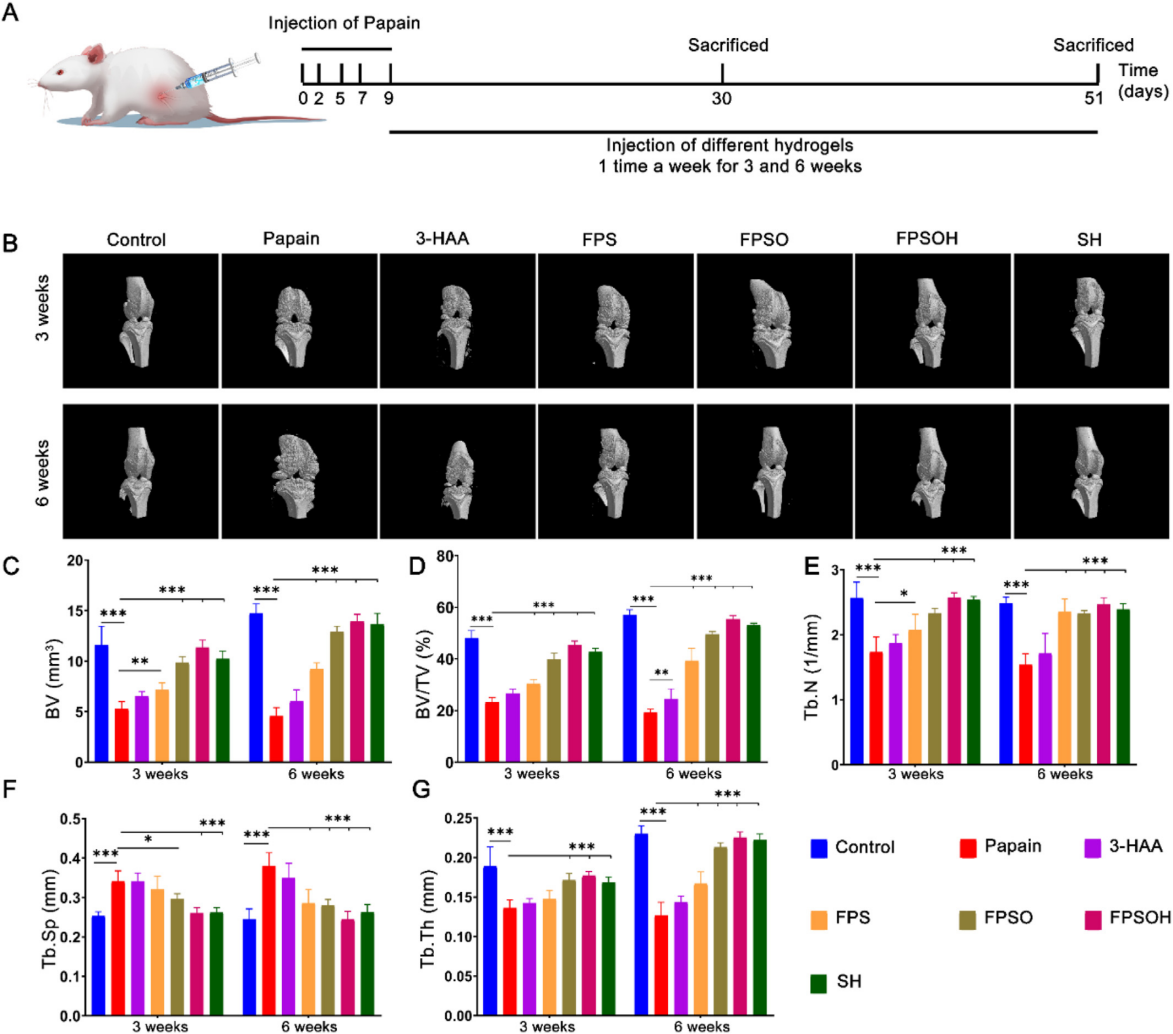
**Evaluation of therapeutic effect of FPSOH on papain-induced osteoarthritis *in vivo*.** (A) Schematic diagram of papain-induced osteoarthritis in Sprague–Dawley (SD) rats. (B) Representative images of the micro-CT scanning of OA with three-dimensional reconstruction at weeks 3 and 6 after papain injection. (C), (D), (E), (F) and (G) Quantitative analysis of BV, BV/TV, Tb·N, Tb. Sp, Tb.Th. ∗P ​< ​0.05; *∗P* ​< ​0.05; *∗∗P* ​< ​0.01; *∗∗∗P* ​< ​0.001.

Rat knee joint specimens were collected in the third and sixth weeks to evaluate the therapeutic effects of the hydrogels (Fig. 5A). Micro-Computed tomography (micro-CT) scans showed the significant manifestations of osteoarthritis in the papain-treated group in the third and sixth weeks compared to the control group (Fig. 5B). Furthermore, the arthritis in the papain-treated group was more severe in on week 6 than that on week 3, manifested by the reduced integrity of the cartilage of the articular surface and increased resorption of the tibial subchondral bone. Meanwhile, the proliferation of osteophytes also increased. In contrast, after treatment, there was a less destruction of the articular surface, less resorption of the subchondral bone, and the formation of osteophytes was suppressed. Based on the micro-CT scans, the group treated with FPSOH hydrogel showed better therapeutic effects than the group treated with SH. Furthermore, the bone volume (BV) of the tibial plateau of the papain-treated group on week 3 was 5.29 ​± ​0.70% mm^3^, which was significantly lower than the 11.60 ​± ​1.86% mm^3^ of the control group. The BV of the papain-treated group (4.58 ​± ​0.80% mm^3^) in the sixth week was lower than that in the third week, indicating that the arthritis became more severe over time. In the third week, the BV of the SH, FPSOH, FPSO, FPS and 3-HAA group were 10.24 ​± ​0.76% mm^3^, 11.35 ​± ​0.73% mm^3^, 9.85 ​± ​0.59% mm^3^, 7.15 ​± ​0.71% mm^3^, 6.45 ​± ​0.46% mm^3^, respectively; in the sixth week they were 13.68 ​± ​1.04% mm^3^, 13.93 ​± ​0.70% mm^3^, 12.91 ​± ​0.54% mm^3^, 9.21 ​± ​0.61% mm^3^, 6.03 ​± ​1.17% mm^3^, respectively. Thus, the FPSOH group showed the highest therapeutic effects, followed by the FPSO, FPS, and the 3-HAA group. Moreover, the FPSPH group showed even better results than those of the SH group. As shown in Fig. 5C–G, additional data on the BV, BV/TV, Tb·N, Tp. Sp and Tb·Th further supported this conclusion. These results indicate that FPSOH hydrogel has a good synergistic therapeutic effect, which performs better than commercial SH.

**Fig. 6:**
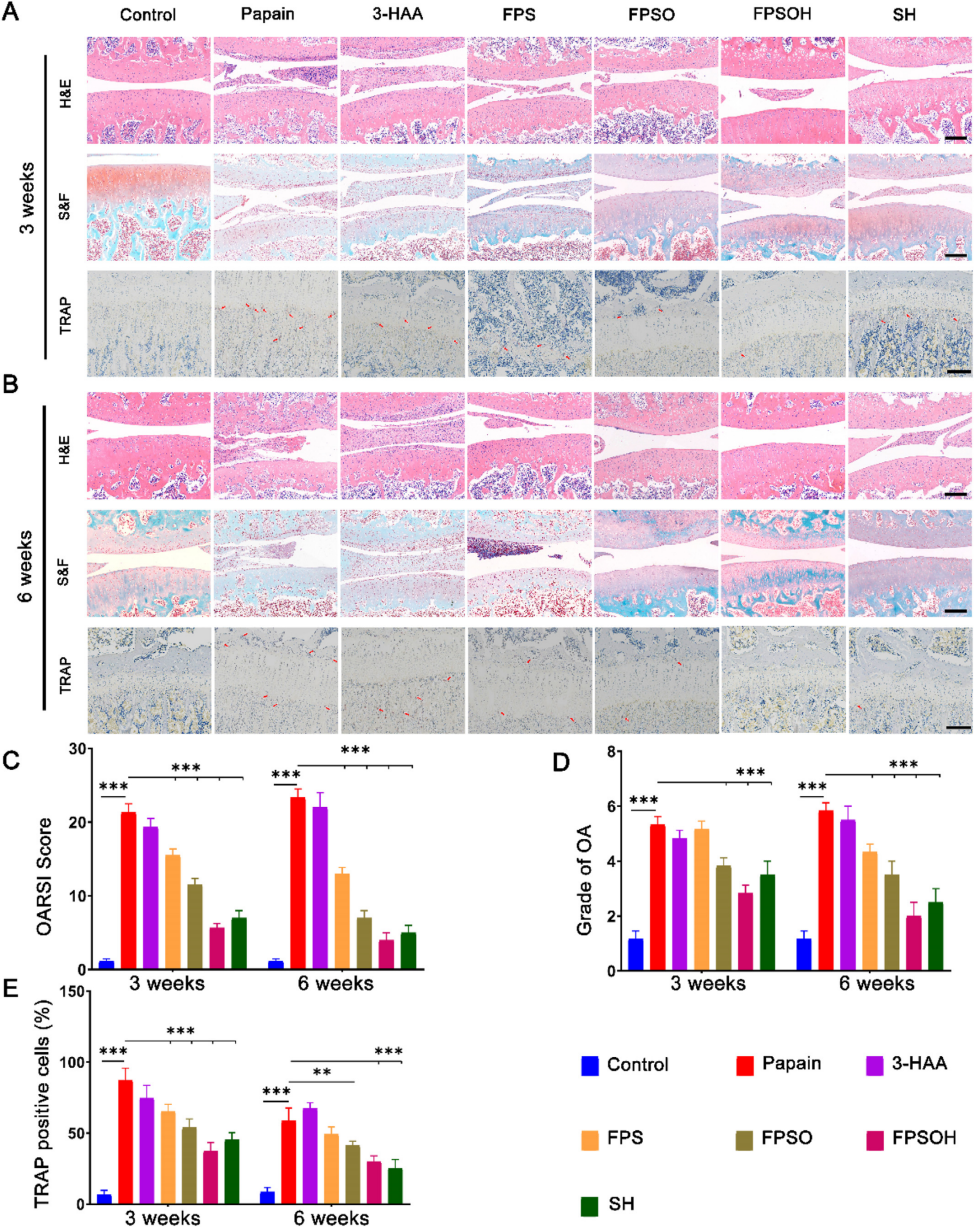
**Staining evaluations of the therapeutic effect of FSPOH on papain-induced OA.** (A) and (B) Representative images of H&E, S&F and TRAP of each group after treatment for 3 and 6 weeks. Scale bar is 200 ​μm. (C) OARSI Score of articular cartilage of each group after treatment for 3 and 6 weeks. (D) Quantitative analysis of the Grade of OA for 3 and 6 weeks. (E) Quantitative analysis of the percentage of TRAP positive cells. *∗P* ​< ​0.05; *∗∗P* ​< ​0.01; *∗∗∗P* ​< ​0.001.

The changes in cartilage morphology were further analyzed with H&E and S&F staining (Fig. 6A–B), the papain-treated group had the characteristics of osteoarthritis. H&E staining revealed the thinning of the cartilage layer, and the surface was no longer flat and showed an increase in the number of vertical fissures, indicating that the cartilage was severely damaged. The S&F staining revealed that as osteoarthritis progressed, the cartilage layer became fibrotic, the collagen matrix degraded significantly, and glycosaminoglycans reduced (stained red). In the papain-treated group, the disease was more severe in the sixth week than that in the third week. After treatment with the different hydrogels, the cartilage surface morphology and S&F staining showed varying degrees of improvement in each group. This improvement was more significant as the treatment time increased, and the therapeutic effect was significantly better in the sixth week than that in the third week. There were more positive cells with S&F staining in the FPSOH group than in the other groups, including the SH group, indicating that FPSOH offered better protection to cartilage. The results of OARSI Score and Grade of OA is illustrated in Fig. 6C–D. All the results further indicated the therapeutic effect of FPSOH on OA. Therefore, the H&E and S&F staining showed that FPSOH inhibits the progression of osteoarthritis and protects articular cartilage from erosion most effectively.

The progression of osteoarthritis includes the pathological processes of chondrocyte apoptosis and degeneration, osteophyte formation, and remodeling of the subchondral bone. The latter occurs because osteoclasts in the subchondral bone are activated. The hyperactive osteoclasts increase the local resorption of the subchondral bone, destroy its structure, and cause it to collapse. Thus, the cartilage loses the underlying supporting and protective structure, which increases the damage to the cartilage and promotes the further development of osteoarthritis. Therefore, TRAP staining was used to examine the effects of hydrogel materials on the osteoclasts in the subchondral bone (Fig. 6A–B and 6E). The number of osteoclasts in the subchondral bone of the papain group was significantly higher than that in the control group and other treatment groups. However, the number of osteoclasts in the FPSOH group was significantly reduced, followed by the SH, FPSO, FPS, and the 3-HAA group. Moreover, on week 6, the number of osteoclasts in each treatment group was significantly lower than that on week 3. This proves that, in addition to protecting chondrocytes, the FPSOH hydrogel can offer indirect protection by inhibiting the activation of osteoclasts in the subchondral bone. These results demonstrate that FPSOH has a protective effect on cartilage and subchondral bone, which inhibits the progression of osteoarthritis. Notably, FPSOH exhibited better therapeutic effects than SH.

**Fig. 7:**
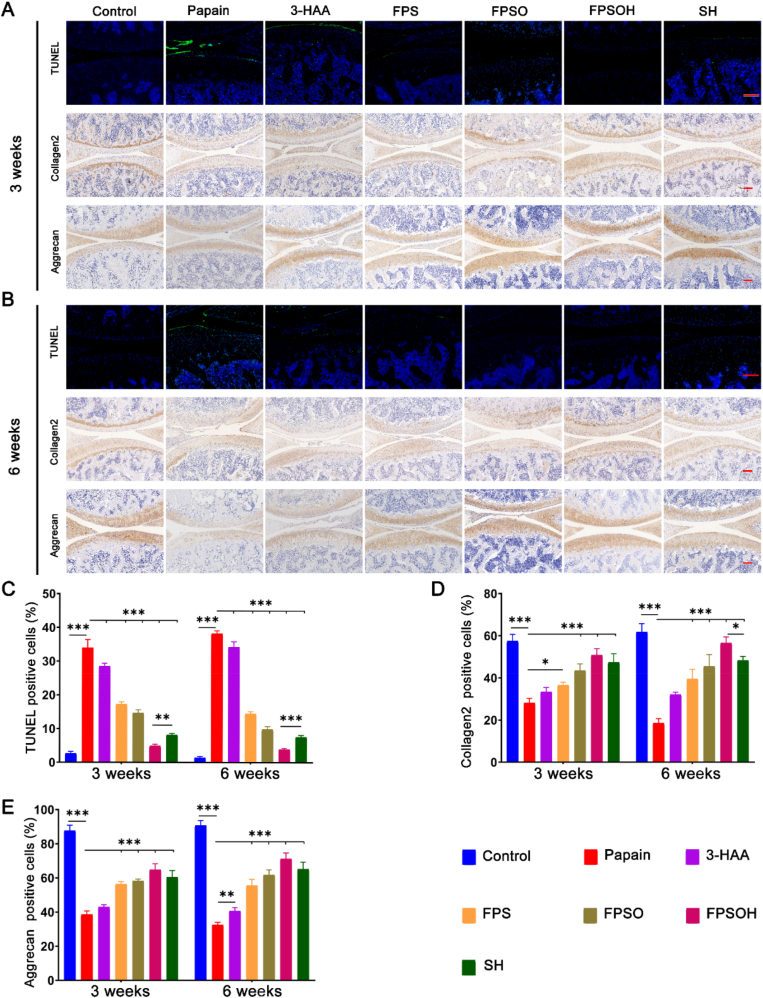
**Staining evaluations of the therapeutic effect of FSPOH on papain-induced OA**. (A) and (B) Representative images of TUNEL immunofluorescence and Collagen2 and Aggrecan immunohistochemistry of each group after treatment for 3 and 6 weeks. Scale bar is 200 ​μm. (C), (D), (E) Quantitative analysis of the percentage of TUNEL and Collagen2 and Aggrecan positive cells. *∗P* ​< ​0.05; *∗∗P* ​< ​0.01; *∗∗∗P* ​< ​0.001.

TUNEL immunofluorescence staining was also used to demonstrate that the hydrogel materials can attenuate the apoptosis of chondrocytes. As shown in Fig. 7A–C, when papain was injected to the joint cavity, the percentage of apoptotic chondrocytes increased significantly compared with that in the control group. This indicates that in the papain-induced osteoarthritis model, oxidative stress occurs, which leads to chondrocyte apoptosis. After treatment, the number of apoptotic chondrocytes was significantly reduced. In addition, the therapeutic effect on week 6 was better than that on week 3, indicating that the FPSOH hydrogel inhibited the progression of oxidative stress in osteoarthritis, thereby reducing chondrocyte apoptosis. The changes in the expression of *collagen2 and aggrecan* were also detected through immunohistochemistry (IHC). In the papain-treated group (Fig. 7A–B and 7D-E), these two indicators had significantly lower percentages of positive IHC results than the control group, and the percentages improved in the treatment groups. This shows that FPSOH have an excellent synergistic therapeutic effect on osteoarthritis. Furthermore, these results show that there was a considerable improvement in osteoarthritis by the sixth week compared to the third week after treatment, indicating that the hydrogel can limit the aggravation of osteoarthritis and hinder its development.

### 3.7 Effects of hydrogel on synovial inflammation and macrophage polarization

In addition to the changes caused by chondrocyte degeneration, osteoarthritis is accompanied by the inflammation of the synovial tissue. Many reports have indicated that there is a strong correlation between synovitis and the progression of osteoarthritis [[6], [7], [8],[10], [11], [12],^29^]. The normal synovial tissue can be divided into an inner synovial layer near the joint cavity and a subsynovial layer. The former is mainly composed of synovial macrophages and fibroblast-like synoviocytes. The activation of synovial macrophages is the most important pathological change associated with synovitis. As synovitis progresses, synovial macrophages are activated into M1 macrophages (pro-inflammatory type), which secrete a large number of inflammatory agents. This further aggravates the inflammation of the synovial membrane, and causes the degeneration of chondrocytes, which aggravates osteoarthritis. Therefore, inhibiting the inflammation of synovial tissue by suppressing the activation of M1 macrophages while inducing the transformation to M2 macrophages (anti-inflammatory type) will not only suppress synovitis but also indirectly alleviate its promotional effect on the progression of osteoarthritis. Therefore, the effects of the hydrogel materials on synovial inflammation and macrophage polarization were investigated.

**Fig. 8:**
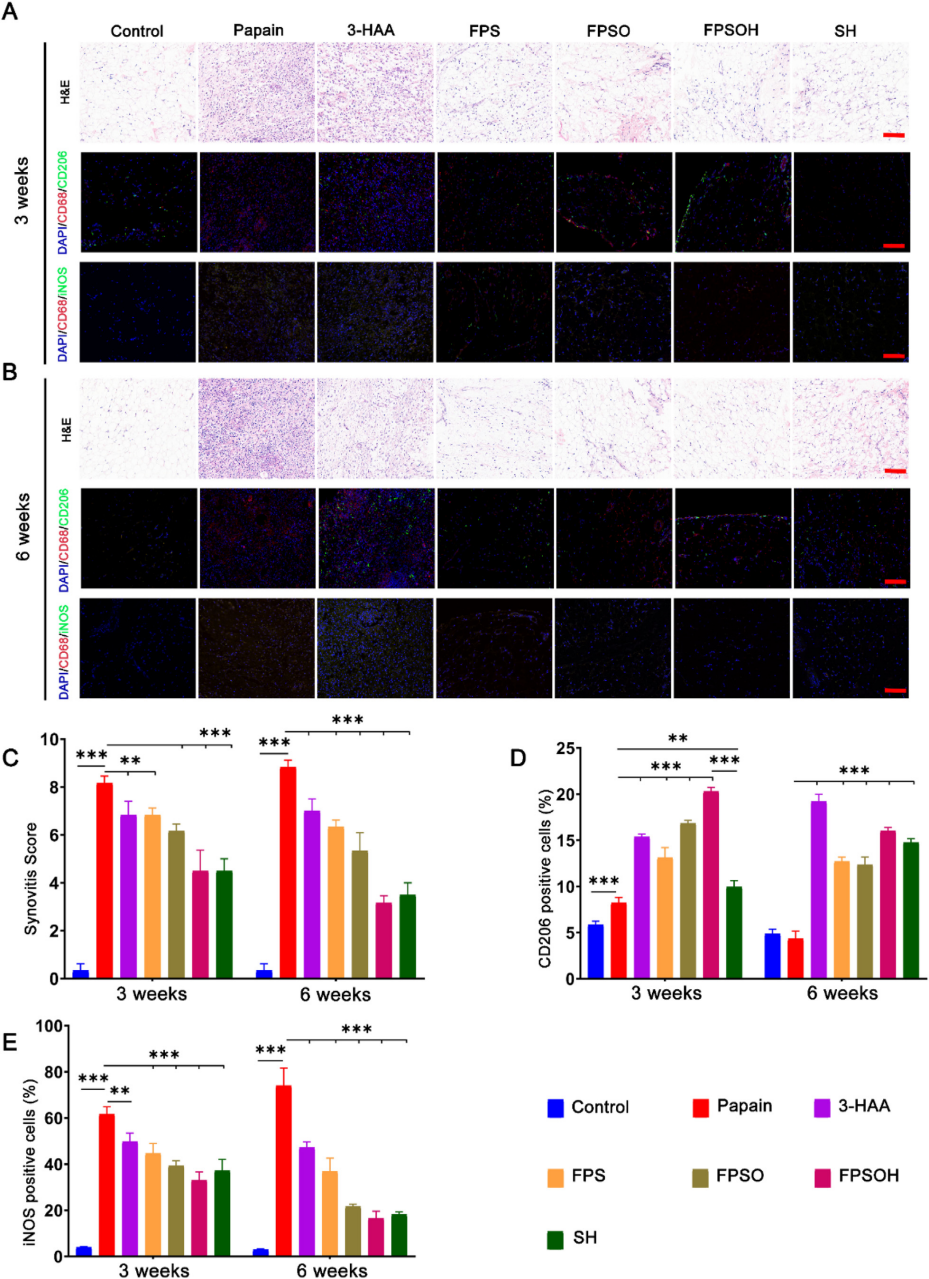
**Staining evaluations of the therapeutic effect of FSPOH on synovitis.** (A) and (B) Representative images of H&E, and immunostaining of iNOS and CD206 of each group after treatment for 3 and 6 weeks. Scale bar is 100 ​μm. (C) Quantitative analysis of the Synovitis Score. (D) and (E) Quantitative analysis of the percentage of M1 and M2 type macrophages. *∗P* ​< ​0.05; *∗∗P* ​< ​0.01; *∗∗∗P* ​< ​0.001.

Fig. 8A–C shows that the synovial tissue in the papain-induced osteoarthritis group showed substantial inflammatory cell infiltration, and the synovial cells exhibited a disordered arrangement and high proliferation on week 3, compared with controls. On week 6, the inflammation of the synovial membrane was further aggravated. However, in the treatment groups, synovitis was suppressed to varying degrees after three weeks. In particular, the synovitis in the FPSOH and SH group was less severe than that in the FPSO, FPS and the 3-HAA group. Notably, the synovitis was less severe in the FPSOH group than that in the SH group. These results show that FPSOH had the highest therapeutic effect than the other group. In addition, on week 6 the severity of synovitis in each treatment group was also lower than that on week 3, indicating that the hydrogel materials can inhibit the progression of synovitis.

The accumulation and phenotypic characteristics of macrophages in synovial tissue with osteoarthritis were identified using immunofluorescence staining (Fig. 8A–B and 8D-E). There were significantly more cells positive for CD68 (synovial macrophage-specific marker) and iNOS (M1 macrophage marker) in the synovial tissue of the papain-induced osteoarthritis group than in the synovial tissue of the control group. In contrast, there was a significant reduction in the number of cells with positive results for CD68 and CD206 (M2 macrophage markers) in the synovial tissue with osteoarthritis. Between the third and sixth weeks, the number of cells positive for CD68 and iNOS gradually decreased in all the treatment groups, and the cells positive for CD68 and CD206 gradually became dominant. The therapeutic effect of FPSOH was better than that of sodium hyaluronate, followed by the FPSO, FPS and the 3-HAA group. Moreover, as the treatment time increased, the regulatory effect on the polarization of synovial macrophages became more significant. These data indicate that the FPSOH can inhibit the inflammation of synovial tissue and regulate the phenotype of synovial macrophages. In addition, the therapeutic effect of FPSOH is better than that of SH gel.

## 4 Conclusions

In this work, a multifunctional injectable FPSOH matrixgel was developed for treating osteoarthritis. FPSOH matrixgel possesses the inherent anti-inflammatory, anti-oxidation stress, *anti*-metalloproteinase, immune regulation and anti-synovitis bioactivity. The anti-inflammatory and antioxidant effects inhibited the progression of osteoarthritis and the development of synovitis. FPSOH hydrogel can reprogram the synovial macrophages from the M1 phenotype to M2 phenotype, and inhibit the synovial inflammation through the NFκB pathway. FPSOH hydrogel can effectively inhibit the initiation and progression of osteoarthritis, protect the cartilage, and inhibit bone remodeling and osteophyte formation. Furthermore, FPSOH can inhibit the initiation and progression of synovitis through its immunosuppressive effect and hinder the promotional effect of synovitis on osteoarthritis. In general, the FPSOH exhibits a good inhibitory effect on the development of osteoarthritis and synovitis through immunoregulation, providing a dual therapeutic effect for osteoarthritis.

## Author statement

**Xinlin Jia**: Conceptualization, Methodology, Data curation, Investigation, Writing – original draft. **Junping Ma**: Conceptualization, Methodology, Data curation, Investigation, Writing – original draft. **Xuzhuo Chen**: Methodology, Investigation. **Wentao Li**: Methodology, Formal analysis. **Xianhao Zhou**: Methodology, Formal analysis. **Bo Lei**: Writing – review & editing, Supervision. **Xin Zhao**: Project administration, Supervision. **Yuanqing Mao**: Writing – review & editing, Supervision, Project administration, Funding acquisition.

## Declaration of competing interest

The authors declare that they have no known competing financial interests or personal relationships that could have appeared to influence the work reported in this paper.
