# Supplementary material for: Immunoregulation and anti-metalloproteinase bioactive injectable polysalicylate matrixgel for efficiently treating osteoarthritis
Source: Mater Today Bio. 2022 May 6;15:100277. doi: 10.1016/j.mtbio.2022.100277 (PMC9114689; doi:10.1016/j.mtbio.2022.100277)
Supplement: Multimedia component 1 [file mmc1.docx]

**Immunoregulation and Anti-metalloproteinase Bioactive Injectable Polysalicylate Matrixgel for Efficiently Treating Osteoarthritis**

Xinlin Jia ^a#^, Junping Ma ^b#^, Xuzhuo Chen ^c^, Wentao Li ^a^, Xianhao Zhou ^a^, Bo Lei ^b^, Xin Zhao ^a^*, Yuanqing Mao ^a^*

*^a^ Shanghai Key Laboratory of Orthopedic Implant, Department of Orthopedic Surgery, Shanghai Ninth People's Hospital, Shanghai Jiao Tong University School of Medicine, Shanghai 200011, China*

*^b^ Key Laboratory of Shaanxi Province for Craniofacial Precision Medicine Research, College of Stomatology, Frontier Institute of Science and Technology, Xi’an Jiaotong University, Xi’an, China*

*^c^ Department of Oral Surgery, Shanghai Ninth People’s Hospital, Shanghai Jiao Tong University School of Medicine, Shanghai Key Laboratory of Stomatology & Shanghai Research Institute of Stomatology National Clinical Research Center of Stomatology, Shanghai 200011, China*

#X. Jia. and J. Ma. contributed equally to this work

*Corresponding author

Xin Zhao, zhaoxinmlg@126.com

Yuanqing Mao, [yuanqingmao@163.com](mailto:yuanqingmao@163.com)

**Keywords**: Bioactive materials; multifunctional hydrogel; tissue engineering; osteoarthritis;

**1. Materials and methods**

**1.1 Materials**

1,1-diphenyl-2-picrylhydrazyl (DPPH) was bought from J&K Scientific. Phosphate buffered saline (PBS), Dulbecco's Modified Eagle Medium (DMEM), penicillin and streptomycin were purchased from Invitrogen. RAW 264.7 macrophages were purchased from ATCC (Manassas, VA). All chemicals were used as received.

**1.2 Rheological and multifunctional properties evaluation**

A TA rheometer was employed to explore the rheological property of FPS, FPSO, and FPSOH hydrogels. Specifically, the storage modulus (G′) and loss modulus (G″) of hydrogels were measured with temperature changes from 4 to 38℃ to verify the thermal sensitivity of hydrogels. Additionally, the self-healing ability of hydrogels were evaluated by measuring G′ and G″ for 5 times with 1%-1000%-1% oscillation strain at 37℃ and 10 rad/s angular frequency. The change of the viscosity of hydrogels with the shear rate from 0.1 to 100 1/s was detected at 37℃ to demonstrate the shear thinning property of hydrogels. In vitro antioxidant capability of hydrogels was evaluated through the DPPH method. The FPS, FPSO, FPSOH and VC were dispersed in 100 mM DPPH methanol solution, and then incubated at 37℃ for 30 min. Thereafter, the absorbance of the solution was detected by the Nanodrop (Schatzbogen 52 D-81829 Munich, Germany) at 514 nm. After that, the color of the solutions was recorded by a camera.

The biodegradation of FPS, FPSO, and FPSOH hydrogels was assessed by recording changes in the weight of the hydrogels placed in PBS at different pH values 7.4 and 5.5 at different times. In short, 0.2 mL different hydrogels were placed into a 24-well plate and 20 μL PBS was added to the plate. At a predetermined point time, the hydrogels were taken out and then the excess water in hydrogel was absorbed by a filter paper. Finally, the hydrogels were weighted and the weight loss percentage was calculated.

Transwell chamber was used to study the release trend of 3-HAA from FPSOH hydrogel in PBS solution with different pH. That is, 0.2 mL of different hydrogels was placed in a 24-well transwell chamber, and 1 mL of PBS was added to the bottom of the well. At a predetermined point time, the absorbance of PBS at the bottom of the well at 316 nm was tested, and the release amount of 3-HAA was calculated by standard curve method.

**1.3 Isolation of primary rat chondrocytes**

The cartilage cap from the femoral head of a three-week-old Sprague Dawley (SD) rat was removed and cut into 1 mm^3^ pieces. The pieces were then digested in a 0.2% Type II collagenase solution prepared with Dulbecco's modified eagle medium/nutrient mixture F-12 (DMEM/F12) medium in an incubator containing 5% CO_2_ at 37 °C for 6 h. The solution was then centrifuged, and the supernatant was removed. The discrete chondrocytes at the bottom of the tube were collected and inoculated into a cell culture dish, which was cultured in DMEM/F12 medium with 10% fetal bovine serum (FBS) and 1% penicillin/streptomycin (100 U mL^-1^) in an incubator containing 5% CO_2_ at 37 °C, according to the standard cell culture method. To maintain the phenotypes of the chondrocytes, the chondrocytes were used for this experiment within three generations.

**1.4 Live-dead cell staining**

Primary rat chondrocytes were cultivated on a six-well plate on a round coverslip with 50,000 cells per well. On the second day after the cells adhered to the wall, 20 μL of the hydrogel materials at different concentrations were placed on the upper layer of the Transwell chamber and incubated with the chondrocytes. On the third day, the numbers of living and dead cells were observed under a confocal microscope (Leica TCS-SP5, DM6000-CFS) according to the instructions accompanying the Calcein/PI Cell Viability/Cytotoxicity Assay Kit (Beyotime Biotechnology, China). According to the instructions, live cells show green fluorescence with Calcein AM staining, while dead cells show red fluorescence with propidium iodide (PI) staining.

**1.5 RT-qPCR test**

In the presence of LPS (100 ng mL^-1^) and IFN-γ(20 ng mL^-1^), RAW264.7 cells were co-cultured with the hydrogel for 24 h, and their RNA was extracted for the detection of inflammatory genes. In addition, 100 µM tert-butyl hydrogen peroxide (TBHB) was added, and the primary rat chondrocytes were co-cultured with the hydrogel for 6 h. Their RNA was extracted for the detection of cartilage metabolism-related genes. The Axygen RNA Miniprep Kit (Axygen, Union City, CA, USA) was used to extract the total RNA, and the Prime Script^TM^ RT Master Kit (Takara, Japan) was used for reverse transcription to obtain cDNA from the RNA template. Subsequently, the TB Green Premix Ex Taq^TM^ Kit (Takara, Japan) was used to perform quantitative real time PCR (RT-qPCR) tests. The primers used in this assay are listed in **Table S5** in the Supplementary Material. All the data were normalized using glyceraldehyde 3-phosphate dehydrogenase (GAPDH) mRNA expression and analyzed using 2^-△△CT^.

**1.6 Macrophage polarization state detection by immunofluorescence**

RAW264.7 cells were pretreated for 24 h according to their treatment group, and then they were stimulated by LPS and IFN-γ for 24 h. The cells were then fixed, permeabilized and blocked, and incubated with the primary antibody and corresponding secondary antibody against iNOS or CD206. Cells with a positive response to iNOS were identified as M1 macrophages, and cells with positive response to CD206 were identified as M2 macrophages.

**1.7 Changes in macrophage polarization detected by flow cytometry**

RAW264.7 cells were seeded on a six-well plate at a density of 100,000 cells per well, and the pretreatments were applied to each group for 24 h. Then, the samples were stimulated with LPS and IFN-γ for an additional 24 h before the cells were harvested and processed for flow cytometry analysis. The antibodies against CD86-BB700 and CD206-Alexa Fluor 647 (BD, CA, USA) were used to label the M1 and M2 macrophages, respectively. The cells positive for CD86 were identified as M1 macrophages, and the cells negative for CD86 and positive cells for CD206 were identified as M2 macrophages. The samples were analyzed using a FACScan flow cytometer (BD, CA, USA).

**1.8 Western blotting (WB) test**

RAW264.7 cells were seeded in a six-well plate at a density of 600,000 cells per well. Each group was pretreated for 24 h, then stimulated by LPS and IFN-γ for an additional 24 h. Different treatments were applied, and then the proteins were extracted. Primary rat chondrocytes were also seeded on a six-well plate at a density of 600,000 cells per well. Subsequently, under the conditions of co-cultivation with each treatment group, 100 μM of tert-butyl hydroperoxide was added to stimulate the chondrocytes for 4 h, and then the proteins were extracted. For the protein extraction, the cells were washed twice with 1X PBS, and the total protein was extracted with radioimmunoprecipitation assay (RIPA) buffer (Beyotime Biotechnoloy, China) according to the instructions. The protein extract was electrophoresed with sodium dodecyl sulphate-polyacrylamide gel electrophoresis (SDS-PAGE), separated, transferred to a 0.22 μm polyvinylidene fluoride (PVDF) membrane, and blocked with 5% skimmed milk at room temperature for 1 h. The protein extract was then incubated overnight with primary antibodies in a refrigerator at 4 °C. The protein extract was then incubated with the secondary antibody at room temperature for 1 h, and image scanning was performed using an Odyssey V3.0 imager (Li-COR. Inc., Lincoln, NE, USA).

**1.9 Detecting the capacity of hydrogels to remove ROS from chondrocytes**

The ROS Assay Kit (Beyotime Biotechnology, China) and DCFH-DA probe were used to detect changes in the ROS, which reflect the capacities of the hydrogels to remove ROS and can be used to evaluate their antioxidation function. The chondrocytes were seeded on the slides of a six-well plate. On the second day, the chondrocytes were co-cultured with the different groups of hydrogels in the presence of 100 μM of tert-butyl hydroperoxide for 4 h, washed three times with PBS, and incubated with the DCFH-DA probe for 30 min in the dark at 37 °C. After incubation, the chondrocytes were washed three times with PBS, stained with Hoechst 33342 (Beyotime Biotechnology, China) for 5 min, and checked with a confocal microscope (Leica TCS-SP5, DM6000-CFS). The Image J software (Bethesda, MD, USA) was used to quantify the intensity of the fluorescent signal.

**2.0 Application of flow cytometry for ROS removal and apoptosis analysis**

Based on the method above, the chondrocytes and the hydrogel material were co-cultured in the presence of TBHB for 4 h. The chondrocytes were collected and processed with an ROS detection kit and Annexin V-FITC Apoptosis Detection kit according to their respective instructions. Subsequently, flow cytometry was performed.


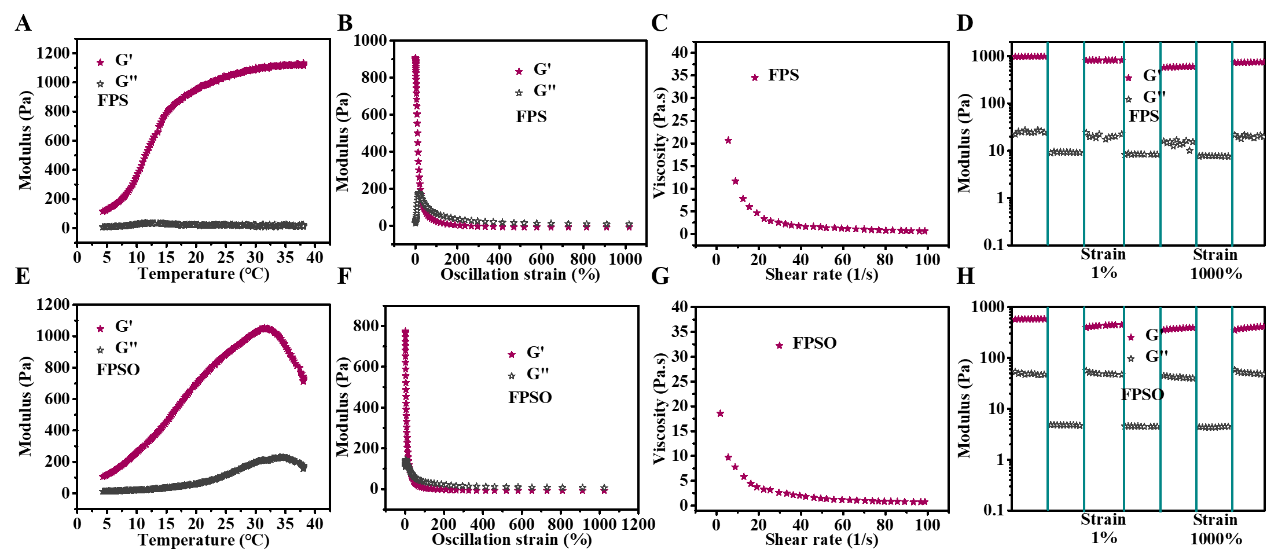


**Figure S1. Rheological characterization of hydrogels.** (A) G′ and G″ with temperature change; (B) G′ and G′′ from 1 to 1000% oscillation strain; (C) Viscosity versus shear rate; (D) G′ and G″ change with cyclic strain of FPS hydrogel; (E) G′ and G″ with temperature change; (F) G′ and G′′ from 1 to 1000% oscillation strain; (G) Viscosity versus shear rate; (H) G′ and G″ change with cyclic strain of FPSO hydrogel.


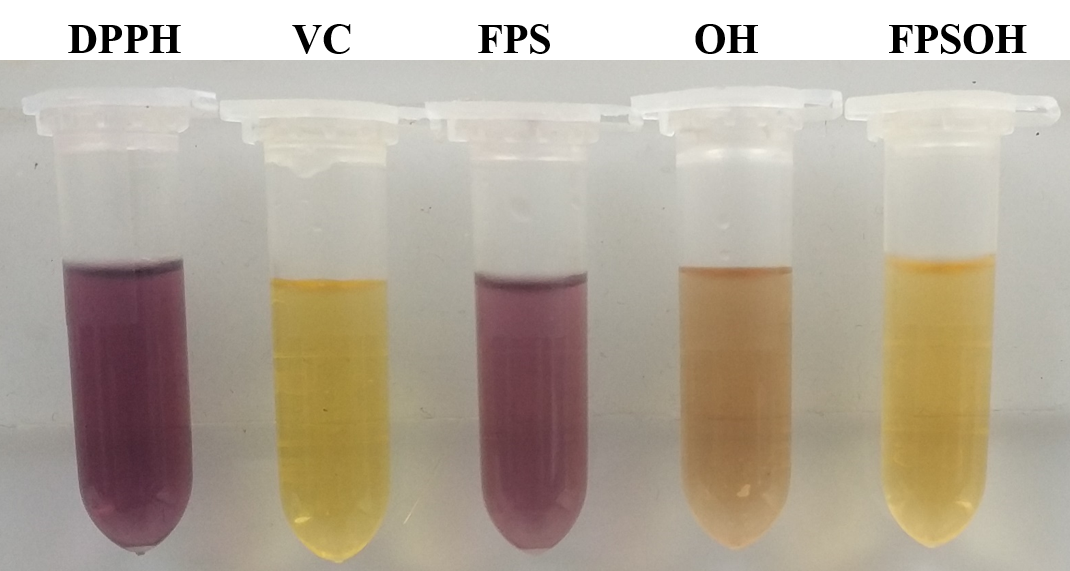


**Figure S2.** Optical photograph of the color change of VC, FPS, OH and FPSOH after co-culture with DPPH for 30 min.


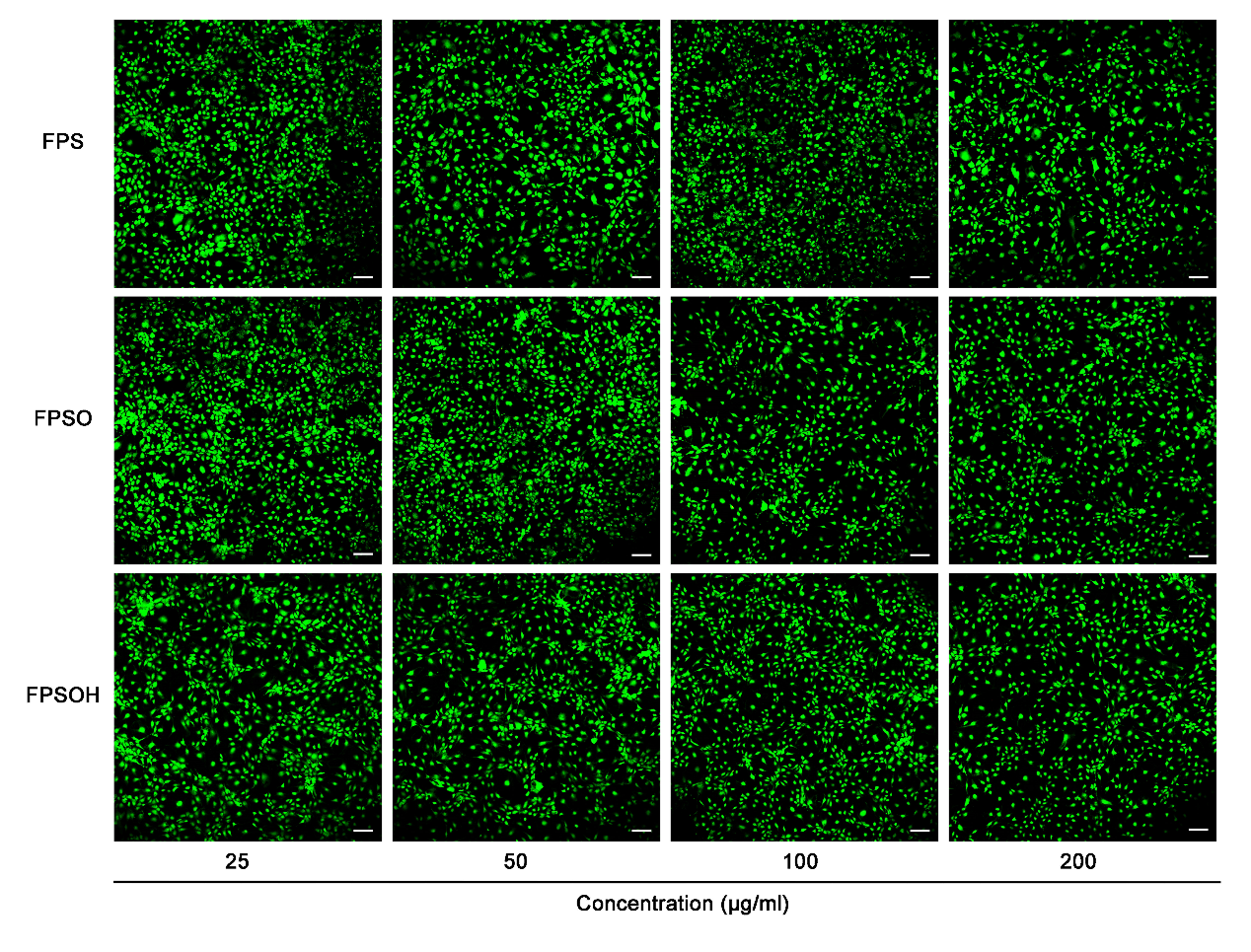


**Figure S3.** The results of live/dead staining about chondrocytes cocultured with FPS, FPSO, FPSOH. Scale Bar is 100 μM.





**Figure S4.** (A) and (B) Flowcytometry analysis of macrophages treated by FPSOH hydrogel in the presence of LPS plus IFN-γ for 24 h. CD86 and CD206 were used as the markers for M1 and M2 phenotype, respectively.


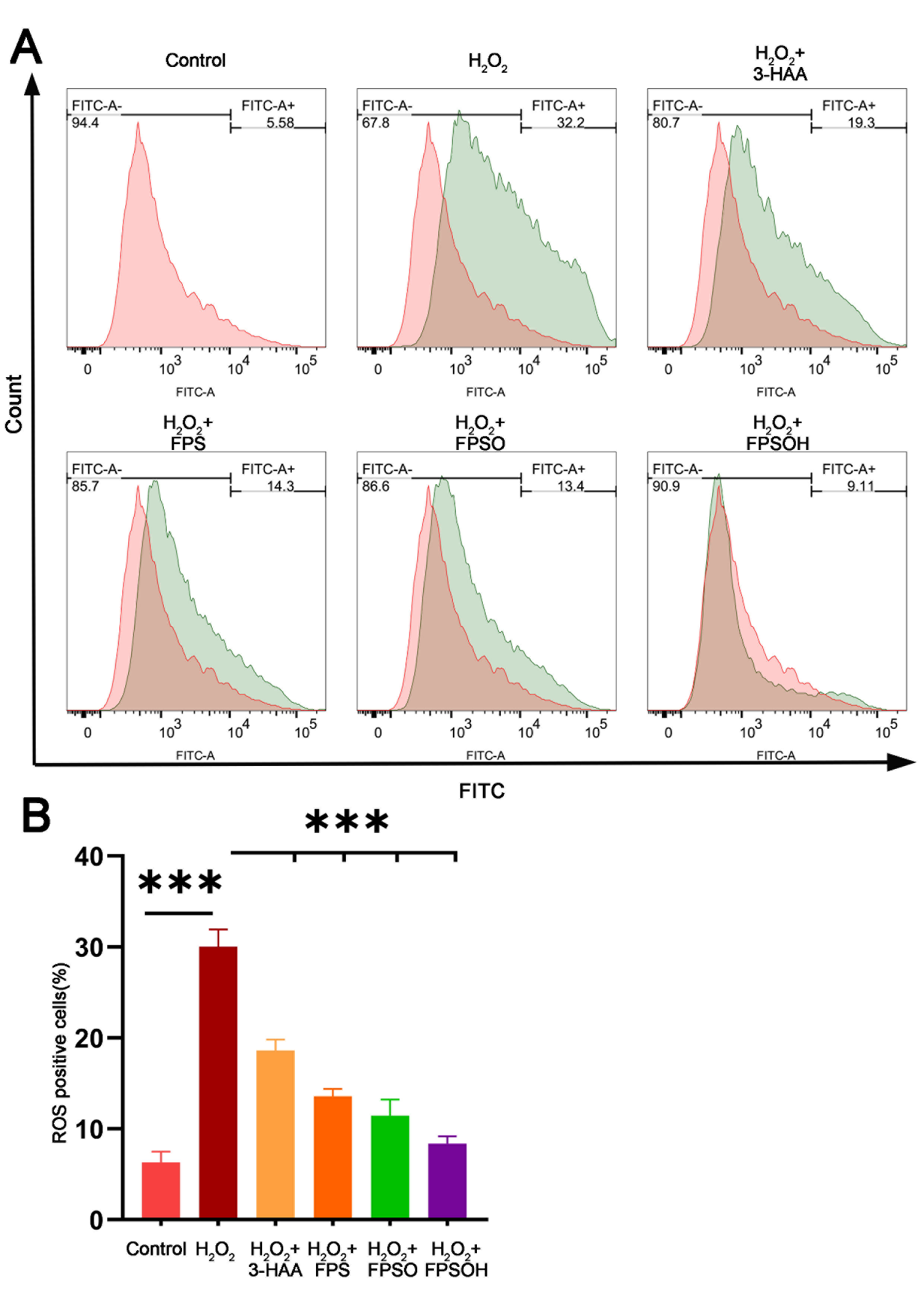


**Figure S5.** Effect of FPSOH on antioxidative stress by Flow cytometry. *P<0.05; **P<0.01; ***P<0.001.


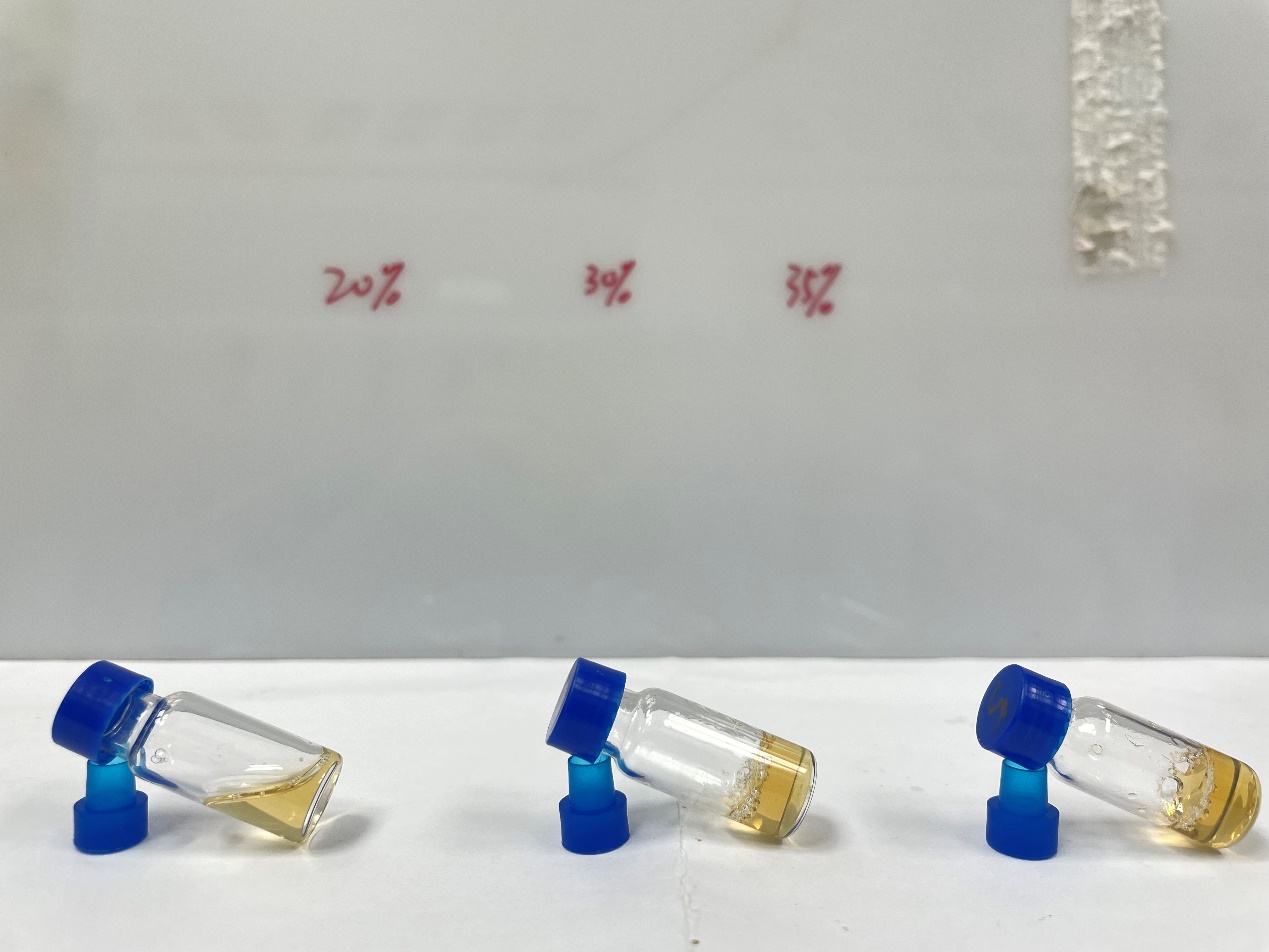


**Figure S6.** Results of gelling properties at different concentrations at 37℃.


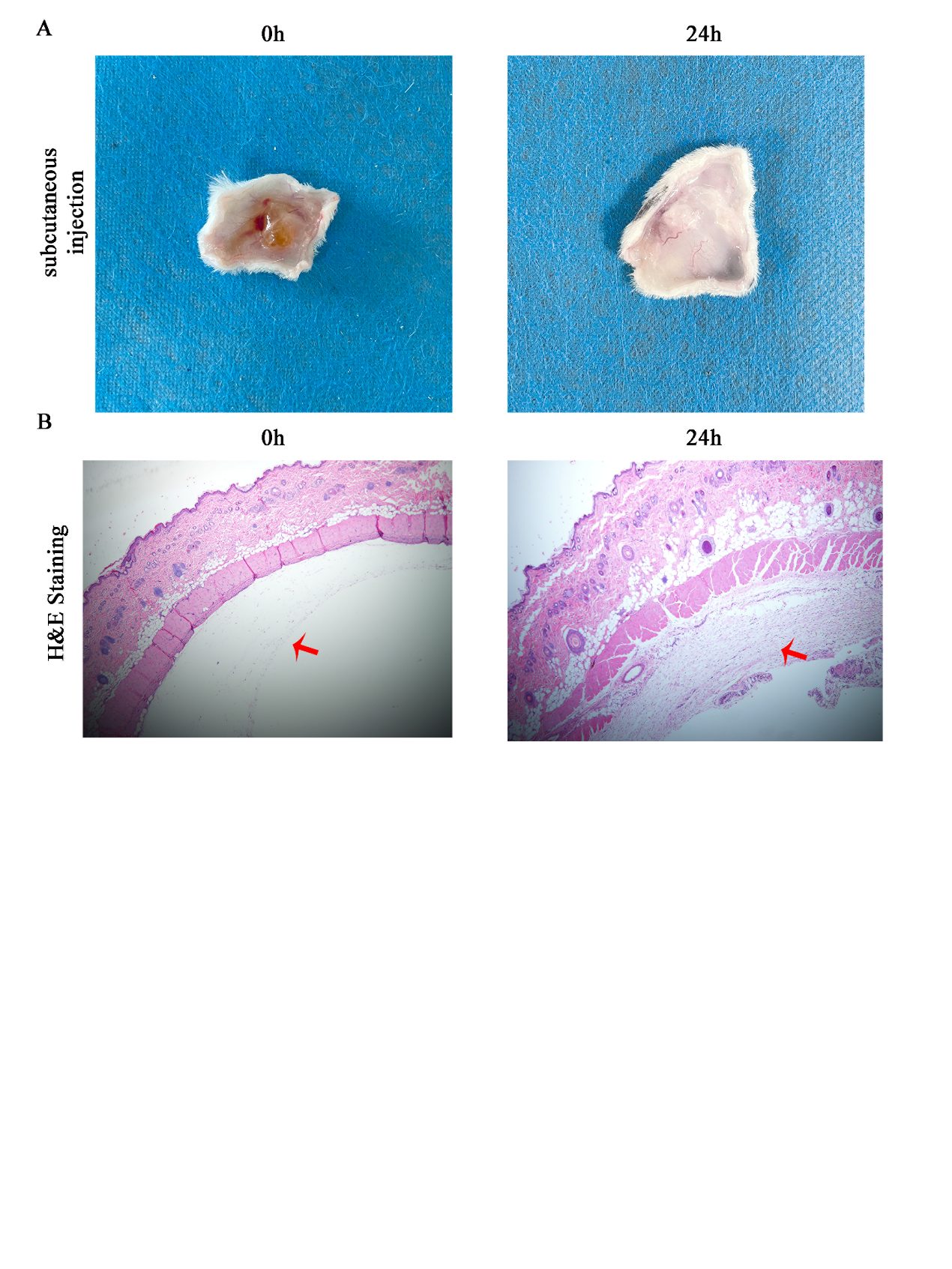


**Figure S7.** H&E staining of skin after the subcutaneous injection of FPSOH hydrogel at 0h and 24h.

**Table S5. The primer used in this study**

| **Primer** | **Sequence** |
| --- | --- |
| **IL-6 Forward** | 5’-CTTCTTGGGACTGATGCTGGTGAC-3’ |
| **IL-6 Reverse** | 5’-AGGTCTGTTGGGAGTGGTATCCTC-3’ |
| **IL-12 Forward** | 5’-CATTGAACTGGCGTTGGAAGCAC-3’ |
| **IL-12 Reverse** | 5’-GGGCGGGTCTGGTTTGATGATG-3’ |
| **IL-1β Forward** | 5’-TCGCAGCAGCACATCAACAAGAG-3’ |
| **IL-1β Reverse** | 5’-AGGTCCACGGGAAAGACACAGG-3’ |
| **TNF-α Forward** | 5’-GCCTCTTCTCATTCCTGCTTGTGG-3’ |
| **TNF-α Reverse** | 5’-GTGGTTTGTGAGTGTGAGGGTCTG-3’ |
| **iNOS Forward** | 5’-ACTCAGCCAAGCCCTCACCTAC-3’ |
| **iNOS Reverse** | 5’-TCCAATCTCTGCCTATCCGTCTCG-3’ |
| **mGAPDH Forward** | 5’-ACCCAGAAGACTGTGGATGG-3’ |
| **mGAPDH Reverse** | 5’-CACATTGGGGGTAGGAACAC-3’ |
| **Aggrecan Forward** | 5’-CGAGTGAACAGCATCTACCAAG-3’ |
| **Aggrecan Reverse** | 5’-GCTCTGTAGTGGAACACAATGC-3’ |
| **Collagen2a1 Forward** | 5’-AGAGCAAGGAGAAGAAGCACAT-3’ |
| **Collagen2a1 Reverse** | 5’-TGGACAGTAGACGGAGGAAAGT-3’ |
| **SOX-9 Forward** | 5’-GTCGGTGAAGAATGGGCAAG-3’ |
| **SOX-9 Reverse** | 5’-ACCCTGAGATTGCCCGGA-3’ |
| **MMP-9 Forward** | 5’-CTGCGTATTTCCATTCATC-3’ |
| **MMP-9 Reverse** | 5’-CCTTGGGTCAGGTTTAGAG-3’ |
| **MMP-13 Forward** | 5’-CAAGCAGCTCCAAAGGCTAC-3’ |
| **MMP-13 Reverse** | 5’-GGCTTTTGCCAGTGTAGGT-3’ |
| **Collagen 10 Forward** | 5’-GAATCGAACGGCCTCTAC-3’ |
| **Collagen 10 Reverse** | 5’-GGATTAGGATGAGCTTGACAG-3’ |
| **rGAPDH Forward** | 5’-TATCGGACGCCTGGTTAC-3’ |
| **rGAPDH Reverse** | 5’-TGCTGACAATCTTGAGGGA-3’ |
